# Fluorescent probes for investigating the internalisation and action of bioorthogonal ruthenium catalysts within Gram-positive bacteria†

**DOI:** 10.1039/d4cb00187g

**Published:** 2024-10-15

**Authors:** Nicole Schubert, James W. Southwell, Melissa Vázquez-Hernández, Svenja Wortmann, Sylvia Schloeglmann, Anne-Kathrin Duhme-Klair, Patrick Nuernberger, Julia E. Bandow, Nils Metzler-Nolte

**Affiliations:** a Faculty of Chemistry and Biochemistry, Chair of Inorganic Chemistry I – Bioinorganic Chemistry, Ruhr University Bochum Universitätsstraße 150 44801 Bochum Germany nils.metzler-nolte@rub.de; b Department of Chemistry, University of York, Heslington York YO10 5DD UK; c Faculty of Biology and Biotechnology, Applied Microbiology, Ruhr University Bochum Universitätsstraße 150 44801 Bochum Germany; d Institut für Physikalische und Theoretische Chemie, Universität Regensburg Universitätsstraße 31 93053 Regensburg Germany

## Abstract

Bioorthogonal reactions are extremely useful for the chemical modification of biomolecules, and are already well studied in mammalian cells. In contrast, very little attention has been given to the feasibility of such reactions in bacteria. Herein we report modified coumarin dyes for monitoring the internalisation and activity of bioorthogonal catalysts in the Gram-positive bacterial species *Bacillus subtilis*. Two fluorophores based on 7-aminocoumarin were synthesised and characterised to establish their luminescence properties. The introduction of an allyl carbamate (R_2_N-COOR′) group onto the nitrogen atom of two 7-aminocoumarin derivatives with different solubility led to decreased fluorescence emission intensities and remarkable blue-shifts of the emission maxima. Importantly, this allyl carbamate group could be uncaged by the bioorthogonal, organometallic ruthenium catalyst investigated in this work, to yield the fluorescent product under biologically-relevant conditions. The internalisation of this catalyst was confirmed and quantified by ICP-OES analysis. Investigation of the bacterial cytoplasm and extracellular fractions separately, following incubation of the bacteria with the two caged dyes, facilitated their localisation, as well as that of their uncaged form by catalyst addition. In fact, significant differences were observed, as only the more lipophilic dye was located inside the cells and importantly remained there, seemingly avoiding efflux mechanisms. However, the uncaged form of this dye is not retained, and was found predominantly in the extracellular space. Finally, a range of siderophore-conjugated derivatives of the catalyst were investigated for the same transformations. Even though uptake was observed, albeit less significant than for the non-conjugated version, the fact that similar intracellular reaction rates were observed regardless of the iron content of the medium supports the notion that their uptake is independent of the iron transporters utilised by Gram-positive *Bacillus subtilis* cells.

## Abstract 2

The bioorthogonal, intracellular Ru-catalyzed deprotection reaction of coumarin dyes is studied inside Gram-positive bacteria.

## Graphical abstract

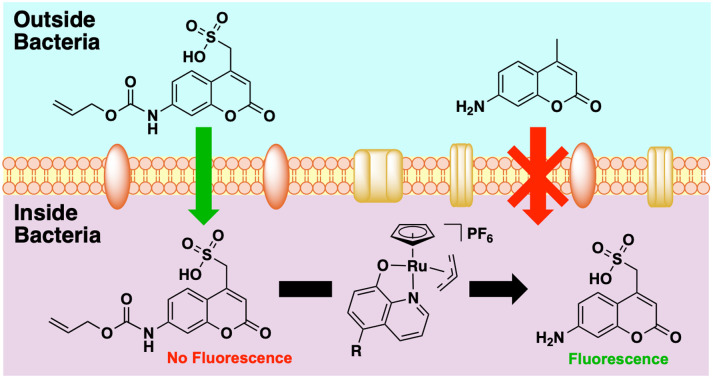

## Introduction

Over the last 15 years, bioorthogonal reactions have become a powerful tool for the chemical modification of biomolecules inside living systems.^1–3^ In particular, the caging of pharmaceuticals and fluorophores has evolved into an important strategy with numerous applications in chemical biology.^4–10^ The caging of an active pharmaceutical or fluorophore with a protecting group typically results in the masking of function or activity, which can be retrieved in a spatiotemporally-controlled manner through the application of photochemical,^11^ chemical,^12^ or metal ion-based triggers.^13,14^ Amines or hydroxyl motifs are the preferred functional groups for masking or ‘caging’ since they are abundant in biomolecules and synthetic chemistry. The emergence of suitable transition metal catalysts (TMCs) offer an attractive alternative to photochemical or chemical uncaging strategies, enabled by use of electron-rich protecting groups like allyl or propargyl due to their susceptibility to metal-mediated bond-cleavage reactions.^15,16^ However, the protecting group, as well as the organometallic catalyst, have to be bioorthogonal to efficiently enable the desired uncaging reactions inside living systems. To achieve this, the introduced compounds should not disturb the function of biomolecules, the uncaging reaction should be chemoselective so that the system does not interfere with the host organism's biochemistry, and the catalyst must be stable and active under biological conditions.^17^ Within this context, the use of TMCs is challenging due to issues of biocompatibility, poor water-solubility, insufficient stability, and their rapid efflux from living cells.^2^ Nevertheless, successful applications of such catalysts has been reported, ranging from the fluorescent labelling of cells, cell compartments,^18^ and proteins,^19^ to the activation of cytotoxic agents,^2,20–22^ or enzyme rescue by the uncaging of tyrosine residues.^23^ The applications of TMCs that are suitable for bioorthogonal reactions have been summarised in several reviews in recent years.^24–34^ Within this arena, ruthenium and palladium have been the most commonly explored transition metals, however more recently, copper and gold have attracted increased attention.^16^

**Fig. 1:**
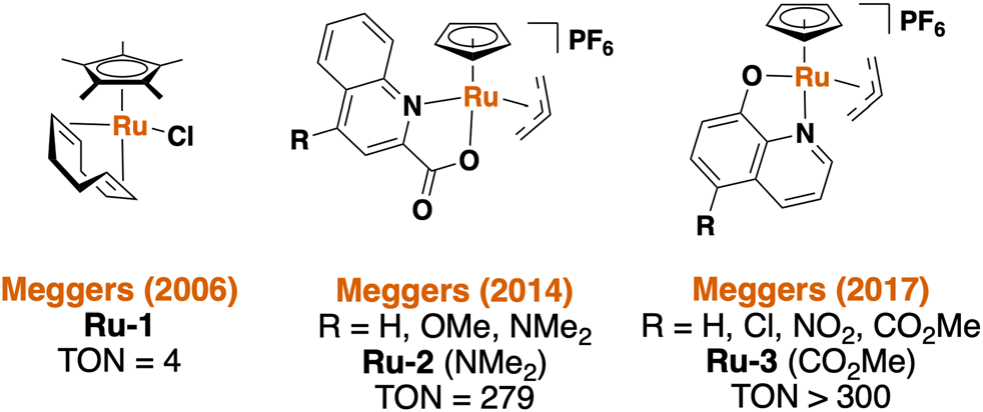
Overview of the ruthenium-based bioorthogonal catalysts published by Meggers *et al.*^1,2,20,36^

Meggers and coworkers are pioneers in the field of bioorthogonal ruthenium-based catalysis, and a summary of the catalysts employed by Meggers *et al.* is shown in Fig. 1. These catalysts are capable of cleaving allyl carbamates to yield the respective primary amines under biologically-relevant conditions (*e.g.* in water, at 37 °C, and in the presence of excess thiols). In 2017, Meggers *et al.* reported a series of bioorthogonal Ru catalysts that achieved the highest turnover numbers (TON) reported thus far.^20^ To this end, a caged non-fluorescent derivative of the well-known fluorophore rhodamine 110 was used in living HeLa cells to demonstrate the successful metal-mediated uncaging reaction with Ru3 (Fig. 1).^20^ In 2023, Southwell *et al.* reported the development of related catalysts linked to siderophore-based targeting molecules for the activation of antibacterial prodrugs within Gram-negative bacteria, such as *E. coli*.^35^ The unmodified organometallic Ru catalyst Ru3, however, has only been applied to mammalian cells, and none of the catalysts shown in Fig. 1 have previously been used in Gram-positive bacteria.

In this study, we investigate whether both the catalyst Ru3 and caged coumarin fluorophores can be taken up by Gram-positive bacteria with the aim of providing evidence of bioorthogonal uncaging within living Gram-positive bacterial cells. Addressing this question is highly important, with regards to the development of much needed new antibiotics, since the bacterial cell envelope prevents many small molecules from entering. In addition, many bacteria actively remove small molecules by expressing efflux-pumps, a key mechanism in developing resistance.^37,38^ Therefore, we have also investigated the dynamics of this system, by monitoring the diffusion of the dyes from the cell.

## Results and discussion

### Catalyst selection, synthesis, and solution dynamics

The synthetic route to the catalyst, Ru3, is shown in the ESI,† (Scheme S1).^20^ The proposed catalytic cycle reported by Meggers *et al.* in 2014 suggested that the Ru^II^ intermediate, generated by the reaction of the catalyst with nucleophiles, such as glutathione and water, to be the active component.^2^ There now exist concerns in the literature regarding the stability of this species. Whilst initially suspected by Waymouth and Kiesewetter *et al.* in 2010,^39^ more recent work by Southwell *et al.* in 2023^35^ and Baiyoumy *et al.* in 2024,^40^ have attributed catalyst decomposition to the reaction of the Ru^II^ species with molecular oxygen. Whilst investigations to this end are not included in this work, the generation of the intermediate species was confirmed by ^1^H NMR kinetics experiments, Fig. S4 (ESI†).

### Selection of fluorophores and their photophysical characterisation

**Fig. 2:**
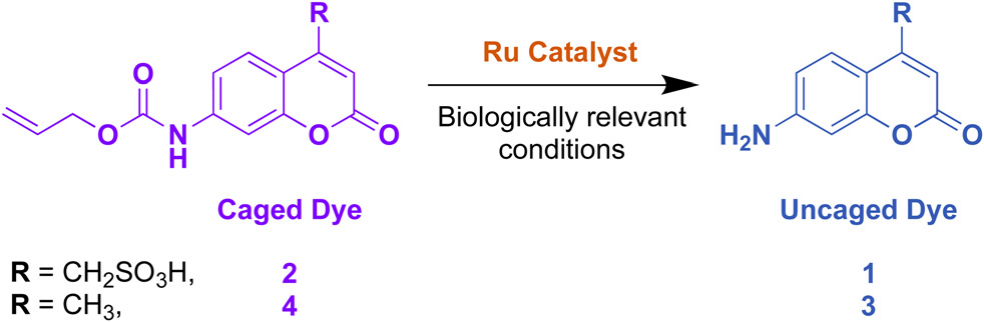
Chemical structure of the caged hydrophilic, 2, and lipophilic, 4, coumarin dyes and their reaction with the ruthenium catalyst under biologically-relevant conditions to form their respective uncaged versions, 1 and 3.

For performing uncaging reactions in Gram-positive bacteria, two different fluorescent dyes based on a coumarin scaffold were selected as substrates. 7-Aminocoumarin-4-methanesulfonic acid, 1, was selected because of its good water solubility and 7-amino-4-methylcoumarin, 3, (also known as coumarin 120), because of its more lipophilic character, and therefore anticipated improved permeation of bacterial membrane. The caged forms of each dye, 1 and 3, were synthesised to form 2 and 4, respectively. The chemical structure of the dyes and the catalyst-mediated uncaging reaction of each dye to release its fluorescent version is summarised below, Fig. 2.

**Fig. 3:**
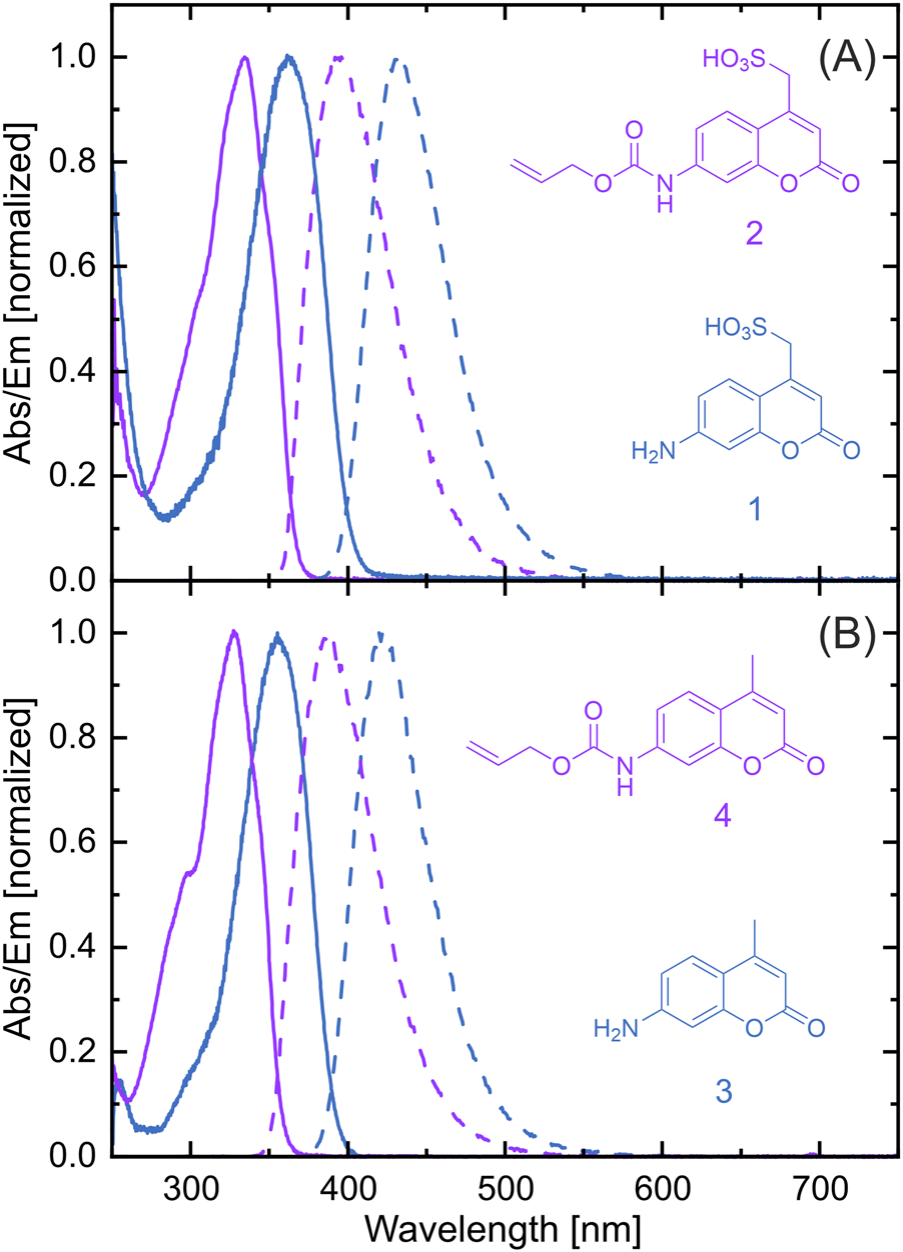
Steady-state absorption (solid lines) and emission spectra (dashed lines) of (A) 7-aminocoumarin-4-methanesulfonic acid dyes (1 and 2), (B) 7-amino-4-methylcoumarin derivatives (3 and 4), dissolved in DMSO.

Coumarin dyes in general, and coumarin 120 (3) in particular, have been investigated in great detail, both experimentally and theoretically.^41–49^ Due to the tuneability of their photochemical properties, for example by means of substitution pattern^50–52^ or solvent choice,^45,53–55^ this class of dye is very versatile and thus used in a wide range of applications.^56–60^ As reported in the literature, the addition of a substituent at the amine moiety leads to a significant spectral blue-shift of the absorption and emission bands,^45,61,62^ which agrees with our observations, Fig. 3. Additionally, coumarin dyes are known to have a pronounced Stokes shift, sensitive to the polarity and viscosity of the solvent environment, which thus influences the photochemical behaviour. The exact Stokes shift values of the dyes used in our work are summarised, Table 1. 7-Aminocoumarin dyes can also exhibit very high emission quantum yields *Φ*_em_ in polar solvents, while non-polar solvents and variations in the substitution pattern, especially at the amino group, lead to a decreased emission quantum yield,^50,51,63^ owing to the highly polar excited state.^56^ Some studies used caged coumarin dyes for biological applications and reported a significant decrease in the *Φ*_em_ values (1–10%) compared to their uncaged analogs,^50^ since a decrease in conformational restriction opens up competing deactivation pathways. We determined the fluorescence quantum yields and found that alloc caging results in a significantly decreased quenching effect in comparison to other substitution patterns,^50^ albeit a clear reduction with regard to the quantum yields of the unprotected compounds is evident, Table 1. Both the non-radiative and radiative decay mechanisms of coumarin 120 (3) in various solvents were previously investigated, unveiling a mono-exponential emission decay in polar solvents, such as DMSO.^41,45^ We applied time-resolved emission spectroscopy using a pulsed LED as excitation source in combination with time-correlated single-photon counting (TCSPC) to determine the emission decay constants. The measured decay traces and the corresponding fitted data are shown in Fig. S3, in the ESI.† In analogy to reports on coumarin 120, 3, all studied molecules show mono-exponential emission behaviour in DMSO (fitted emission lifetimes are summarised in Table 1).

**Table 1:** Summary of emission wavelengths, Stokes shifts calculated from the steady-state absorption and emission maxima, Emission lifetimes *τ* detected with a TCSPC apparatus, and the corresponding emission quantum yield *Φ*_em_ determined with an integrating sphere of all studied dyes dissolved in DMSO

Substrate	Max. emission wavelength [nm]	Stokes shift, Δ*ν* [× 10^3^ cm^−1^]	Emission lifetime, *τ* [ns]	Emission quantum yield, *Φ*_em_
1	434	4.64	3.6	0.95
2	391	4.60	1.7	0.71
3	422	4.47	3.3	0.98
4	388	4.62	1.3	0.53

### *In vitro* activity of the catalyst

The significant spectral blue-shift of the emission maximum of the caged dye allowed its uncaging to be quantified by measuring the fluorescence intensity at the emission maximum. *In vitro* cleavage of the alloc group of 2 and 4 by Ru3, at three different catalyst loadings, to form 1 and 3, respectively, was studied under biological conditions in Belitzky minimal medium (BMM) at 37 °C, in the presence of air and excess of glutathione (see Table 3 in the Expt. section for composition of the medium). All experiments used freshly prepared catalyst stock solutions, to minimise decomposition. The TON and conversion for each reaction was measured after 4 h, Table 2. This timeframe was chosen since maximum catalytic conversion is reached by this point (Fig. S10, ESI†) and the stability studies for related catalysts reported by Southwell *et al.* suggested that catalyst decomposition is less significant over this timeframe.^35^ Results reveal linear correlations for both caged substrates, where conversions are lower for 4. The calculated TONs are lower than those reported by Meggers *et al.*, which is attributed to the lower concentration of the substrates used in our studies, and the known oxygen sensitivity of the catalyst.^20,35,40^ For this work, it was important to determine TON values at substrate concentrations and under conditions that mimic those to be used in our subsequent bacterial assays.

**Table 2:** Conversions and TONs obtained using different equivalents of Ru3 (2.5, 5 or 10 mol% *vs.* dye) for the uncaging reactions of 2 and 4 to form 1 and 3 respectively, in chemically defined medium (BMM). Dye concentrations were around 40 μg mL^−1^

Dye	Equivalents of Ru3/mol%					
	2.5		5		10	
	Conversion/%	TON	Conversion/%	TON	Conversion/%	TON
2	42	21	65	16	85	11
4	9	7	14	6	23	5

### Effect of Ru-catalysts and dyes on *B. subtilis*

Once the uncaging of the fluorescent dyes by Ru3 was established *in vitro*, the growth effects of Ru3 and the dyes had on the Gram-positive model organism *B. subtilis*, were investigated over the same timeframe. To evaluate this, the *B. subtilis* cultures were exposed to the respective sample, over various concentrations during early exponential growth. The growth of the treated cultures was monitored by measuring the optical density of the cultures at 500 nm (OD_500_). Growth of *B. subtilis* was inhibited by the catalyst in a concentration-dependent manner (Fig. S12, ESI†), while no growth inhibition was observed for the fluorophores over the tested concentration range (Fig. S13, ESI†). A Ru3 concentration of 1 μg mL^−1^ was chosen for future experiments as it still allowed significant bacterial growth, whilst maximising the catalyst concentration for the uncaging reactions.

### Ru catalyst internalisation

First, we studied the cellular internalisation of the Ru catalyst using inductively coupled plasma optical emission spectrometry (ICP-OES). To this end, cultures of *B. subtilis* were treated with Ru3 at a concentration of 1 μg mL^−1^ during early exponential growth, then washed twice with buffer to remove extracellular ruthenium. After cell disruption by ultrasonication, the ruthenium content of the crude cell extract was analysed by ICP-OES. The intracellular concentration of ruthenium in cells treated with the catalyst was determined to be 0.88 ± 0.20 μmol mL^−1^ (equalling 89 ± 20 μg mL^−1^). This is about a 90-fold accumulation compared to the Ru concentration in the medium during incubation, and well above the untreated control (0.004 ± 0.007 μmol mL^−1^ equalling 0.4 ± 0.7 μg mL^−1^), indicating that Ru3 is readily taken up by the bacterial cells. This is an observation that, to the best of our knowledge, has not been reported previously.

### Incubation of bacteria with caged dye and subsequent catalyst addition

After establishing suitable experimental conditions in the minimal medium as described above, catalytic uncaging reactions with 2 and 4, to release 1 and 3 respectively, were performed in *B. subtilis* cultures. For these uncaging experiments, the amount of fluorophore was adjusted based on the concentration of 1 μg mL^−1^ of Ru3 and the concentration ratios used for the kinetic uncaging experiments described above. The dyes 1–4 were tested with varied catalyst equivalents: 10 μg mL^−1^ (10 mol%), 25 μg mL^−1^ (4 mol%), and 50 μg mL^−1^ (2 mol%).

**Fig. 4:**
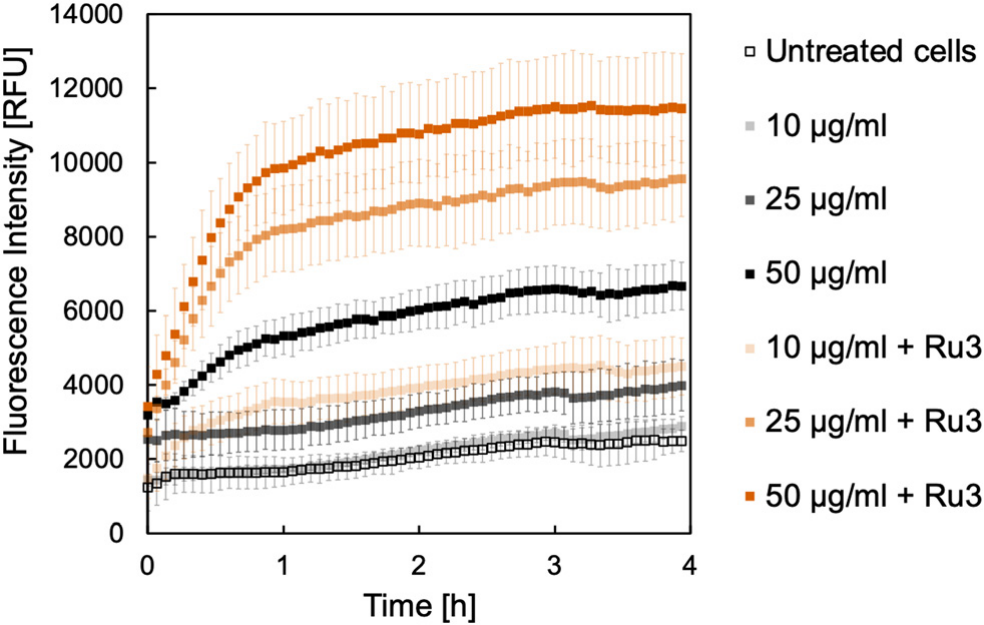
Formation of 7-aminocoumarin-4-methanesulfonic acid, 1 from the corresponding caged coumarin dye 2. The fluorescence intensity was measured at 468 nm in relative fluorescence units [RFU]. *B. subtilis* cultures were incubated with 2 in the following concentrations: 10 μg mL^−1^ (light shade), 25 μg mL^−1^ (medium shade), and 50 μg mL^−1^ (dark shade) without (black) and with Ru3 (orange, 1 μg mL^−1^), as indicated. Untreated cells, without catalyst and dye addition are represented as empty black squares. Error bars are the standard deviations of three independent biological replicates.

**Fig. 5:**
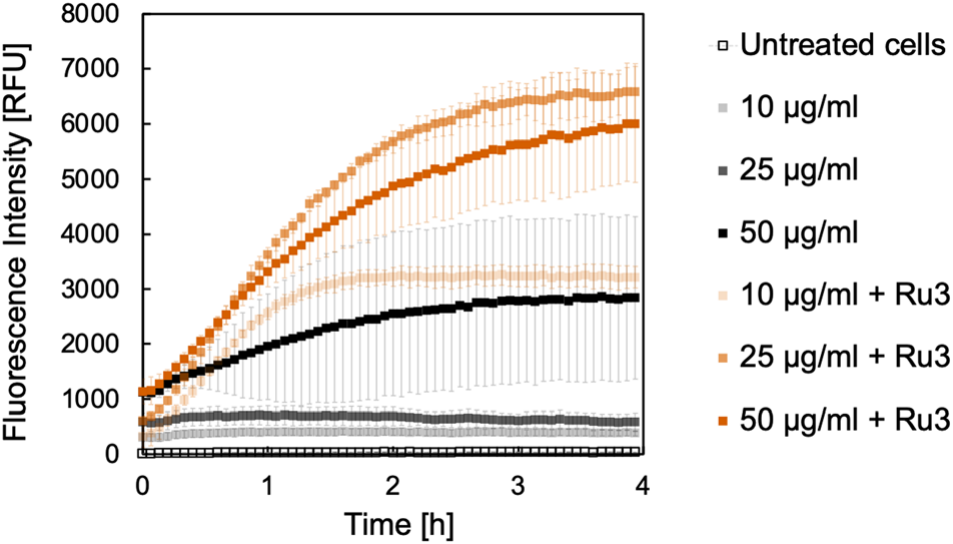
Formation of 7-amino-4-methylcoumarin, 3 from the corresponding caged coumarin dye 4. The fluorescence intensity was measured at 421 nm in relative fluorescence units (RFU). *B. subtilis* cultures were incubated with 4 in the following concentrations: 10 μg mL^−1^ (light shade), 25 μg mL^−1^ (medium shade), and 50 μg mL^−1^ (dark shade) without (black) and with Ru3 (orange, 1 μg mL^−1^), as indicated. Untreated cells, without catalyst and dye addition are represented as empty black squares. Error bars are the standard deviations of three independent biological replicates.

Initially, *B. subtilis* cultures were incubated with the caged fluorophores 2 or 4 at the selected concentrations for 15 min. Cells were then washed and placed into fresh medium before Ru3 was added at a concentration of 1 μg mL^−1^. This way, since Ru-3 internalisation was established, if the uncaging reaction is observed spectroscopically, the result is indicative of dye internalisation. Untreated cultures served as a negative control, wherein a low fluorescence intensity was expected at the tested wavelengths over the course of the experiment. Cultures treated with the caged dye 2 or 4 but without catalyst, served as additional controls, which were likewise expected to show only low fluorescence intensities. The results of the uncaging experiments for 2 and 4 are shown in Fig. 4 and 5, respectively. As expected, the untreated cultures show only low fluorescence intensities, notably with little change in fluorescence over the 4 h time course of the experiment. However, when the catalyst Ru3 was added, a significant increase in fluorescence was observed within the first hour. This increase in fluorescence indicates the formation of the uncaged 7-aminocoumarins. Whilst minimum conversion took place in the absence of Ru3, uncaging was significantly faster and yielded more product with Ru3 at all dye concentrations. It is noteworthy that all curves reach maximum intensity after 4 h. The maximum conversion of the water-soluble caged coumarin derivative 2 is reached after 1 h whereas that of 4 takes significantly longer where maximum conversion is reached after 4 h, for the higher concentrations of 4 (25 and 50 μg mL^−1^). These initial studies are suggestive of dye internalisation however it is important to consider the possibility of diffusion of the caged form out of the cell, on addition of the fresh medium, and subsequent uncaging in the extracellular space.

### Retention of uncaged dyes inside *B. subtilis* cells

**Fig. 6:**
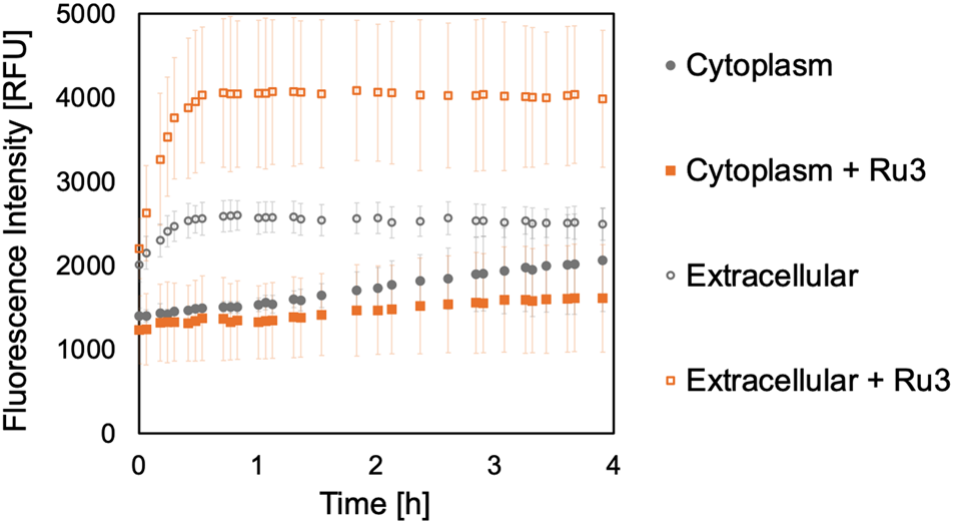
Bioorthogonal uncaging reaction of 2 (50 μg mL^−1^) catalysed by Ru3 (1 μg mL^−1^) addition to either the cytoplasmic or extracellular fractions. Samples were excited at 356 nm and the fluorescence intensity was measured at 468 nm for 1 in relative fluorescence units [RFU]. The error bars are the standard deviations of three independent biological replicates. Since the fluorescence intensity of the untreated culture is negligibly low with a value of 10 RFU, the control is omitted for clarity.

**Fig. 7:**
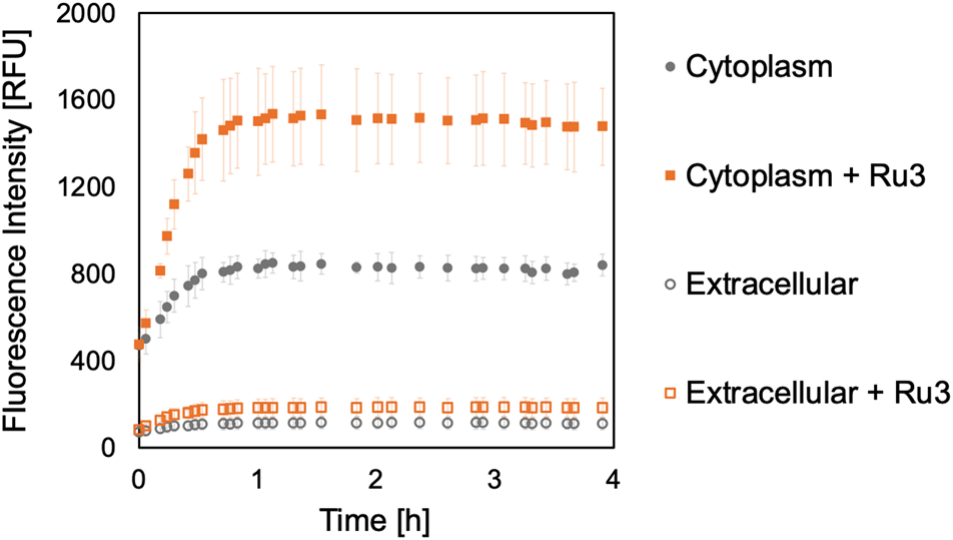
Bioorthogonal uncaging reaction of 4 (50 μg mL^−1^) catalysed by Ru3 (1 μg mL^−1^) addition to either the cytoplasmic or extracellular fractions. Samples were excited at 356 nm and the fluorescence intensity was measured at 421 nm for 3 in relative fluorescence units [RFU]. The error bars are the standard deviations of three independent biological replicates. Since the fluorescence intensity of the untreated culture is negligibly low with a value of 10 RFU, the control is omitted for clarity.

To investigate whether the caged and uncaged dyes remain inside the bacteria, and allow us to more confidently assign intracellular reactivity, additional experiments were performed. For these experiments, *B. subtilis* cultures were again treated with the caged fluorophores 2 or 4 (50 μg mL^−1^) for 15 min. Subsequently, the cells were harvested, washed, and disrupted by ultrasonication, followed by separation of the debris from the soluble cytoplasmic fractions. Whilst the supernatant from the initial incubation was discarded, that from the second washing was collected, representing any release of the caged fluorophores into the extracellular medium during the washing step. Ru3 was then added to the cytoplasmic and extracellular fractions, and fluorescence was measured to detect the uncaged dyes. The addition of Ru3 (1 μg mL^−1^) to the cytoplasmic fractions of cells previously incubated with 2 gave little increased fluorescence signal, especially compared to those without catalyst addition, Fig. 6 (filled symbols). In contrast, the cytoplasmic fraction of cells previously incubated with 4 showed fluorescence attributed to its uncaged form, 3 and a substantial increase for the fraction treated with catalyst due to the increased formation of 3, Fig. 7 (filled symbols). Fluorescence from the extracellular fraction, in the absence of catalyst, can be attributed to dye (caged and uncaged) that has diffused out of the cell. A further fluorescence increase following catalyst addition to the same fraction can be attributed to the amount of uncaged dye produced. The extracellular fraction from cells previously incubated with 2 gave a significant fluorescence increase, especially following catalyst addition, Fig. 6 (empty symbols). Altogether, considering previous dye incubation studies, these results indicate that 2 is initially internalised but does not remain in the cell upon exposure to fresh medium during the washing step. In contrast, the extracellular fraction from cells incubated with 4 showed negligible fluorescence, Fig. 7 (empty symbols) suggesting that 4 is internalised and remains inside the cell, even after washing.

### Localisation of caged dye compared to its uncaged version inside *B. subtilis* cells

**Fig. 8:**
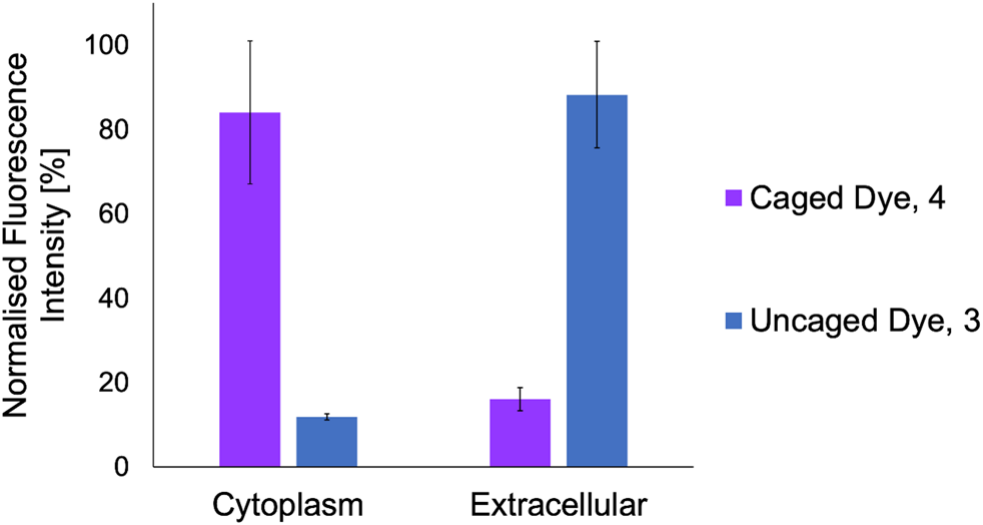
Normalised fluorescence intensity of cytoplasmic fractions and extracellular media following incubation of *B. subtilis* with 4 (50 μg mL^−1^) and addition of Ru3 in DMSO (final concentration of Ru3 1 μg mL^−1^) two hours before harvest. The fluorescence intensities were measured at 392 nm (for the caged dye 4, purple) and 420 nm (for the uncaged dye 3, blue). Data is shown as relative fluorescence intensity [%] of 4 in each fraction (purple) and 3 in each fraction (blue). The error bars are the standard deviations of three independent biological replicates.

Now that the internalisation of Ru3 has been confirmed, as well as that of the more lipophilic dye, 4 and its retention within the bacteria, it was important to compare the retention of the dye's uncaged form, 3. This time, the cells were incubated with 4, then washed thoroughly to remove extracellular dye, after which Ru3 was added two hours prior to their harvest. After 2 h, the cells and their extracellular medium were separated and collected. The cells were disrupted by ultrasonication followed by removal of the debris, and the soluble cytoplasmic fractions were analysed. The fluorescence of the cytoplasmic fractions and extracellular media were measured at 392 nm and normalised to determine the relative localisation of the caged dye, 4Fig. 8 (purple bars). The relative localisation of the uncaged dye, 3 was measured at 420 nm, Fig. 8 (blue bars).

As expected, the caged dye 4 was mainly found in the cytoplasmic fraction, whilst its uncaged version 3 was found predominantly in the extracellular fraction. Since previous studies have shown that the caged dye 4 and catalyst Ru3 are co-located within the bacterial cells, and that formation of the uncaged dye 3 occurs through Ru-catalysed de-allylation, it can be concluded that the bioorthogonal reaction proceeds within the bacteria and that the product, 3, subsequently diffuses out of the cells, or is actively effluxed.

### Incubation of bacteria with caged dye and subsequent addition of various siderophore-conjugated catalysts

The experiments described above were performed on Gram-positive bacteria, which have a relatively simple cell envelope. Work by Southwell *et al.* reported Ru catalysts with covalently attached siderophores for the uncaging of antibacterial drugs in Gram-negative bacteria,^35^ which have an additional protective outer-membrane. Siderophores are small-molecule chelators produced by bacteria that are released into the extracellular environment to selectively bind Fe^III^ for uptake into the cell. The Fe-siderophore complexes are internalised into the bacterium through the different layers of the cell envelope *via* specific, active transporters.^64^

**Fig. 9:**
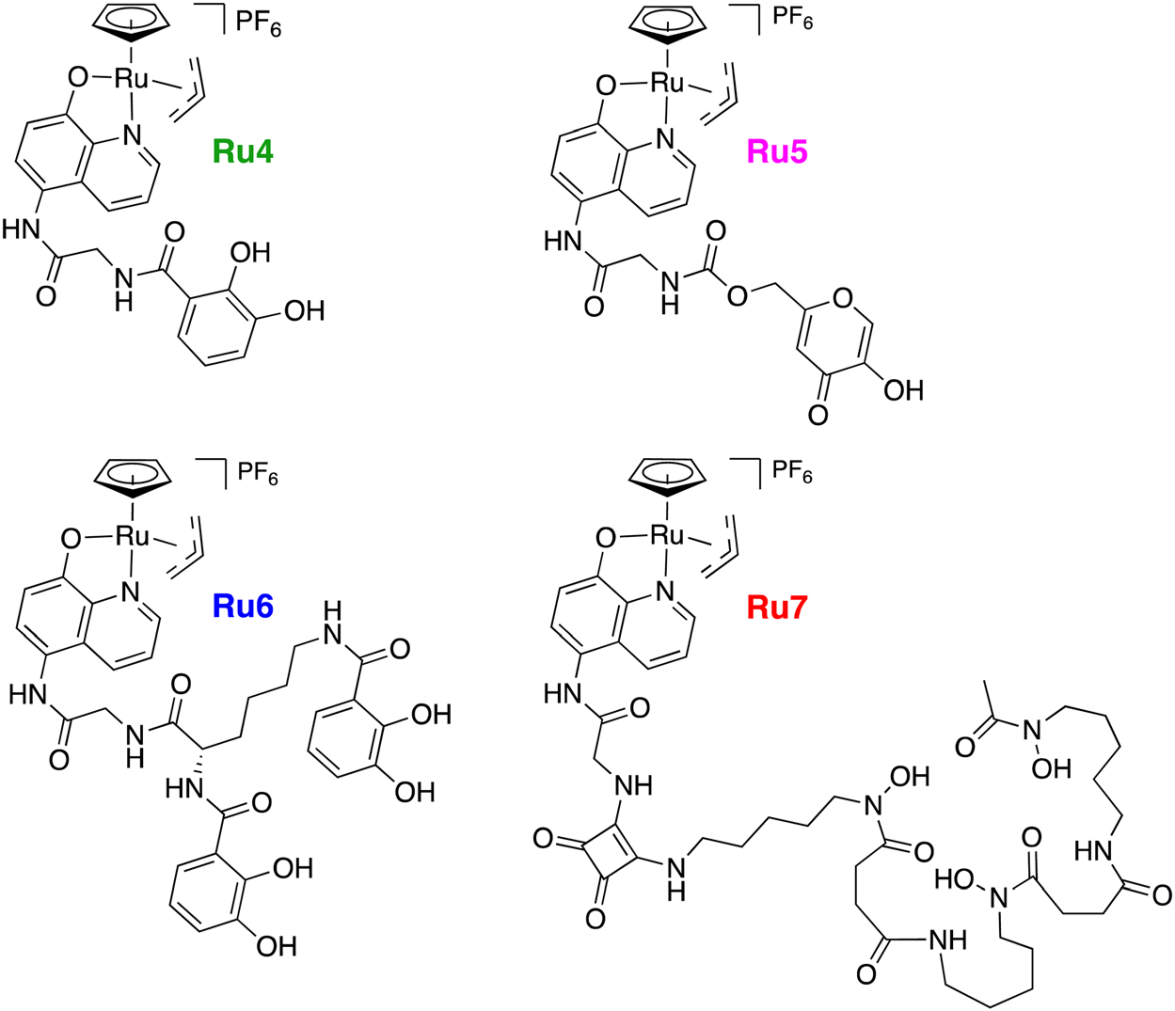
Structures of the siderophore-linked ruthenium catalysts, developed by Southwell *et al.*^35^

In the present work, it was of interest to see whether in *B. subtilis* the uncaging reaction on coumarin dyes described above would also proceed with the previously reported siderophore-linked Ru catalysts Ru4–Ru7, Fig. 9. Since the catechol-based iron chelating units present in Ru4 and Ru6 are also found in endogenous catecholate siderophores used by *B. subtilis*, such as bacillibactin and itoic acid,^65^ siderophore-mediated uptake was conceivable.

**Fig. 10:**
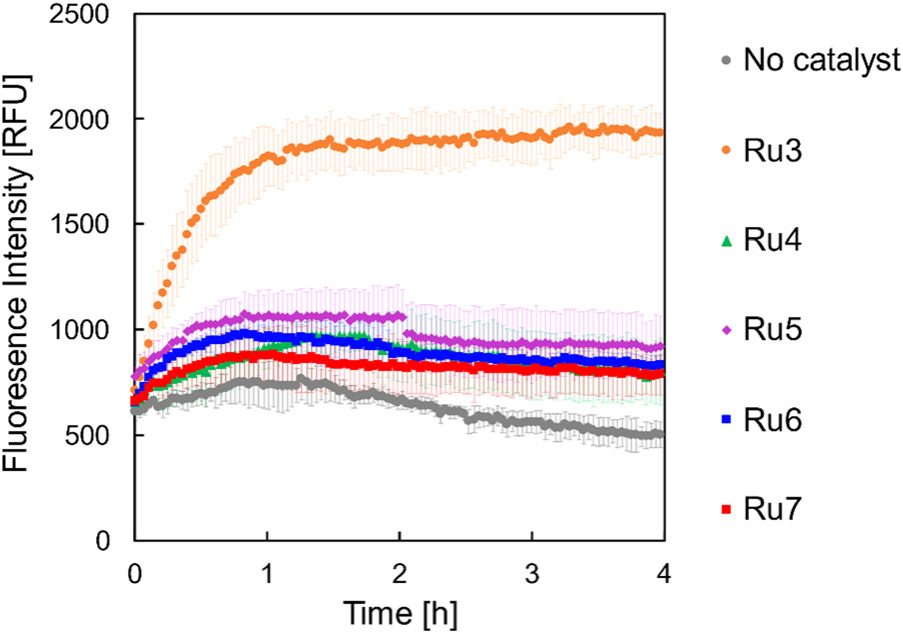
Bioorthogonal uncaging reaction of 4 (50 μg mL^−1^) catalysed by Ru3–Ru7 at (1 μg mL^−1^), under iron-limited conditions. The samples were excited at 356 nm and the fluorescence intensity was measured at 421 nm (emission maximum of the uncaged dye 3) in relative fluorescence units (RFU). Error bars represent the standard deviation of three independent biological replicates.

**Fig. 11:**
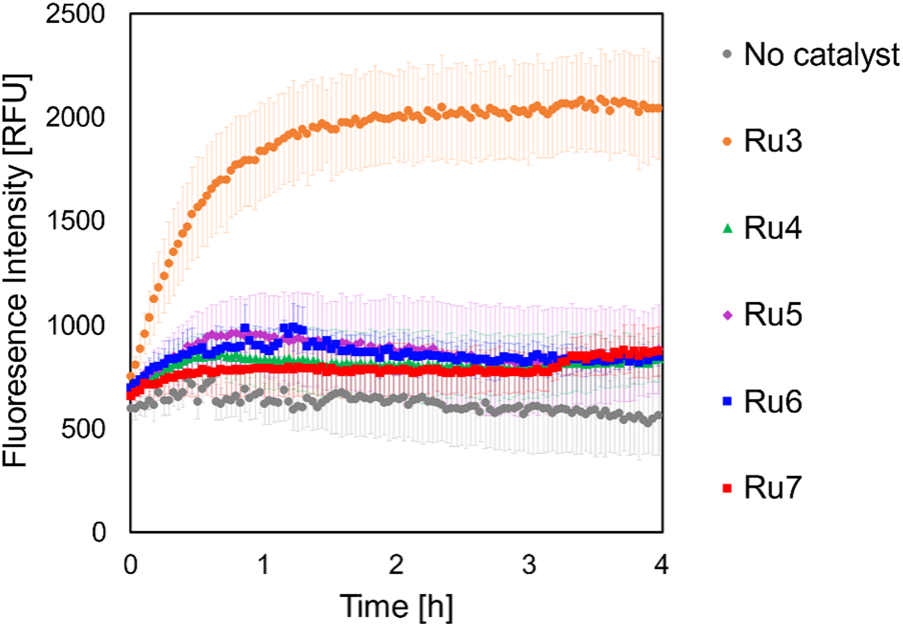
Bioorthogonal uncaging reaction of 4 (50 μg mL^−1^) catalysed by Ru3–Ru7 at (1 μg mL^−1^), under iron-supplemented conditions. The samples were excited at 356 nm and the fluorescence intensity was measured at 421 nm (emission maximum of the uncaged dye 3) in relative fluorescence units (RFU). Error bars represent the standard deviation of three independent biological replicates.

*B. subtilis* cells were grown under iron-limited and -supplemented conditions, to give an indication of whether the catalyst-siderophore conjugates were internalised *via* iron-uptake pathways. Since the use of siderophores by bacteria is upregulated to mitigate low intracellular iron levels, if increased dye uncaging is observed under iron-limited conditions, it is suggestive of uptake by these means.^66–68^ Bacterial cultures were grown to the early exponential growth phase in modified BMM medium under iron-limited and -supplemented conditions, and subsequently incubated with 4. The cultures were then washed, resuspended in fresh medium, and to them was added each of the catalysts Ru3–Ru7 at 1 μg mL^−1^. Fluorescence intensity of the uncaged dye 3 was then monitored over a period of four hours, including controls with no catalyst addition. The results for cells incubated under iron-limited conditions are shown in Fig. 10, and those under iron-supplemented conditions in Fig. 11.

An increase in fluorescence intensity was detected for all catalysts in both iron-limited and -supplemented media, however, by far the steepest increase (4-fold) was observed for Ru3, suggesting the reaction does indeed take place intracellularly. The siderophore-linked catalysts all performed similarly to each other, with approximately 2-fold increases at both iron concentrations. Whilst these results imply that the dye uncaging reaction with siderophore-linked Ru catalysts proceeds to some extent inside *B. subtilis*, the fact that iron-concentration makes no difference is not confirmative of their internalisation *via* siderophore-mediated iron-uptake pathways. Consequently, conjugation of the Ru catalyst to the siderophores investigated in this case impedes their uptake compared to Ru3, whose lower molecular weight and more lipophilic nature probably facilitates passive diffusion across the membrane. The siderophore-conjugated Ru catalysts however might still provide a degree of selectivity, *i.e.* for targeting of bacterial over mammalian cells, but this was not investigated. As expected, low fluorescence intensities were observed for bacteria grown with the caged fluorophore in the absence of any Ru catalyst.

## Summary and conclusion

We have shown for the first time that the bioorthogonal, ruthenium-mediated uncaging of an allyl carbamate to release its respective amine, can be efficiently carried out in Gram-positive bacteria, using *B. subtilis* as a model system. To monitor the uncaging reaction spectroscopically, two caged derivatives of fluorescent coumarin dyes 2 and 4 were synthesised. The uncaging of these dyes by catalyst Ru3 was confirmed under biologically-relevant conditions. Whilst internalisation of the catalyst was confirmed by ICP-OES measurements, that of the caged dyes and their cytoplasmic retention was evaluated using dye incubation experiments. Even though both 7-aminocoumarin-4-methanesulfonic acid 2 and the more lipophilic dye 7-amino-4-methylcoumarin 4 appeared to be internalised, only the latter remained inside the cells or closely associated with them, for at least two hours. Interestingly, its uncaged version, 3, was subsequently released from the cell. As bacteria can prevent small molecules from entering cells and actively export small molecules *via* efflux pumps, it was important to establish whether the caged fluorophores and catalysts could be co-located within bacterial cells at sufficient concentrations and long enough for the uncaging reaction to occur. The different abilities of the dyes to enter and remain inside the bacterial cells underline the importance of choosing the right fluorophore for the investigation of intracellular bioorthogonal uncaging reactions.

Since compound 4 was taken up into and stayed inside cells, we exploited this fact to also evaluate the internalisation of previously-reported siderophore-conjugated versions of the catalyst, Ru4–Ru7, by monitoring uncaged dye formation following bacterial incubation with 4, over 4 h. In contrast to Ru3, the siderophore-conjugated catalysts were poorly taken up into the bacteria. As Ru3 is smaller and more lipophilic, it is hypothesised that uptake is probably facilitated by passive diffusion whereas this is not possible for the larger siderophore-conjugated versions. Additionally, to provide information regarding how the siderophore conjugates were internalised, we monitored the uncaging reaction under iron-limited and iron-supplemented conditions, respectively. Unfortunately, since low activity and no difference in activity between the conditions was observed, we could not positively attribute uptake to the hijacking of siderophore-mediated iron-uptake pathways. We have recently demonstrated the importance of the medium, or more precisely its iron content, for the activation of caged prodrugs of antibacterials in Gram-negative bacteria by investigating the siderophore-linked Ru compounds Ru4–Ru7.^35^ Relatedly, the iron-dependent activity of antimicrobial peptides was demonstrated in elegant work using radioactive Fe isotopes recently, to imply siderophore-mediated uptake.^69^ The present work is more fundamental in that it avoids the use of radioactive tracers or antibiotics (that will influence cell metabolism) and provides the first example of a particular bioorthogonal uncaging reaction inside Gram-positive bacterial cells. The system provided here is robust enough to be used as a basis for future studies for investigating bacterial processes and antibacterial applications where prodrugs are used.

## Experimental

### Materials and methods

All chemicals were of reagent grade quality or better and were obtained from commercial suppliers. The chemicals were used without further purifications. Solvents were used as received. Reactions with dried solvents were carried out using standard Schlenk techniques. ^1^H NMR and ^13^C NMR measurements were performed on a Bruker DPX-200 or a Bruker DRX-400 spectrometer at 298.5 K. Signals were referenced to residual ^1^H or ^13^C signals from the deuterated solvents and the chemical shifts reported in parts per million (ppm). The abbreviations for the peak multiplicities are as follows: s (singlet), d (doublet), dd (doublet of doublets), t (triplet), q (quartet), and m (multiplet). The spectral analysis was performed using the program *MestReNova* (Version 10.0). ESI mass spectra were recorded on a Bruker Esquire 6000 and infrared spectra were recorded on an ATR unit using a Bruker Tensor 27 FTIR spectrophotometer at 4 cm^−1^ resolution. The signal intensity is abbreviated br (broad), s (strong), m (medium), and w (weak).

### Synthesis

The synthesis of the catalyst's ligand except for the first step and the synthesis of the catalyst Ru3 was carried out as published by Meggers *et al.*^20^ The synthesis of the siderophore-linked catalysts Ru4–Ru7 was previously reported by Southwell *et al.*^35^ The synthesis of 7-aminocoumarin-4-methane sulfonic acid was adapted from the literature and the compound was used without further modifications.^70^

#### 8-Hydroxyquinoline-5-carboxylic acid

Acrolein diethyl acetal (12.50 mL, 0.85 g mL^−1^, 81.63 mmol, 2.5 eq.) was dissolved in 250 mL of 1 M HCl and the solution was heated to 50 °C. 3-Amino-4-hydroxybenzoic acid (5 g, 32.65 mmol, 1 eq.) was added to the solution over a period of 30 min. The orange reaction mixture was refluxed for 75 min. After cooling to room temperature, the acidic solution was neutralised using 1 M NaOH, and the desired product precipitated from the neutralised solution. The precipitate was isolated from the solution by filtration, washed with water, and dried in an oil pump vacuum to yield 8-hydroxyquinoline-5-carboxylic acid (6.18 g, 26.43 mmol, 81%) as an orange powder. ^1^H NMR (200 MHz, d_6_-DMSO): *δ*_H_ [ppm] 9.47 (1H, dd, *J* = 10, 2 Hz), 8.90 (1H, dd, *J* = 6, 2 Hz), 8.24 (1H, d, *J* = 8 Hz), 7.69 (1H, dd, *J* = 8, 4 Hz), 7.13 (1H, d, *J* = 8 Hz, H). ^13^C NMR (50.3 MHz, d_6_-DMSO): *δ*_C_ [ppm] 167.8, 157.7, 148.1, 138.2, 134.6, 133.4, 128.1, 123.2, 116.9, 110.1. IR (ATR): *ν̃* [cm^−1^]: 3246 (br), 1681 (s), 1506 (m), 1371 (m), 1265 (m), 1138 (s), 823 (m), 723 (m), 630 (m), 491 (w). ESI-MS (positive mode): *m*/*z* [%] 189.90 ([M + H]^+^, 100%), 229.90 ([M + K]^+^, 25%).

#### 7-((Allyloxy)carbamoyl)amino-coumarin-4-triethylammoniummethansulfonate (2)

7-Amino-4-methanesulfonic acid (0.5 g, 1.96 mmol, 1 eq.) was suspended in a triethylammonium bicarbonate buffer (5 mL, 1 M). The grey suspension was cooled to 0 °C with an ice bath and allyl chloroformate (0.42 mL, 1.13 g mL^−1^, 3.84 mmol, 1.96 eq.) was added dropwise at this temperature. After stirring the suspension for 1 h at 0 °C, the suspension was allowed to warm to ambient temperature and allyl chloroformate (0.42 mL, 1.13 g mL^−1^, 3.84 mmol, 1.96 eq.) was added again carefully. Afterwards, the grey suspension turned into a dark red solution. The reaction mixture was allowed to stir at room temperature for 5 h. Subsequently, the solvent was removed using a lyophilizer and the solid was purified by reverse-phase flash column chromatography (C18, eluent : H_2_O : ACN [1 : 0 → 0 : 1], the crude product was dry-loaded onto Celite). After removal of the solvent, 7-((allyloxy)carbamoyl)amino-coumarin-4-triethylammoniummethansulfonate (0.17 g, 0.49 mmol, 25%) was obtained as slightly yellow solid. *R*_f_ (C18, H_2_O : ACN 1 : 1, detection: UV): 0.74. ^1^H NMR (400 MHz, d_6_-DMSO): *δ*_H_ [ppm] 10.19 (1H, s, NH), 8.89 (1H, s, SO_3_H), 7.84 (1H, d, *J* = 8Hz), 7.54 (1H, s), 7.33 (1H, d, *J* = 8 Hz), 6.24 (1H, s), 6.03–5.94 (1H, m), 5.32 (1H, d, *J* = 16 Hz), 5.26 (1H, d, *J* = 12 Hz), 4.65 (2H, d, *J* = 4 Hz), 3.99 (2H, s), 3.11–3.04 (6H, m), 1.16 (9H, t, *J* = 8 Hz). ^13^C NMR (100.06 MHz, d_6_-DMSO): *δ*_C_ [ppm] 160.4, 154.1, 153.1, 150.0, 142.4, 133.0, 127.8, 118.0, 114.0, 113.9, 113.8, 104.3, 65.1, 53.2, 45.9, 8.7. IR (ATR): *ν̃* [cm^−1^] 3257 (br), 2991 (br), 2704 (br), 1716 (s), 1616 (s), 1573 (m), 1525 (m), 1415 (w), 1394 (m), 1326 (w), 1220 (s), 1184 (s), 1029 (s), 991 (m), 862 (m), 825 (m), 769 (m), 707 (m), 655 (m), 624 (w). ESI MS (negative): *m*/*z* [%] 338.20 ([M − H]^−^, 100%).

#### Allyl (4-methyl)coumarin-7-carbamate (4)

7-Amino-4-methylcoumarin (150 mg, 0.86 mmol, 1 eq.) was dissolved in 10 mL dry pyridine and the solution was cooled to 0 °C with an ice bath. Allyl chloroformate (0.46 mL, 1.13 g mL^−1^, 4.26 mmol, 5 eq.) was added carefully at 0 °C. The reaction mixture was stirred at this temperature for 2 h. After this period, the reaction mixture was warmed to ambient temperature and stirring was continued overnight. Subsequently, the reaction mixture was quenched with 1 M HCl and the resulting aqueous phase was extracted with ethyl acetate three times. The combined organic layers were dried over MgSO_4_, filtered, and concentrated under reduced pressure. The crude product was purified by flash column chromatography (SiO_2_, eluent : pentane : EtOAc [1 : 1], the crude product was dry-loaded onto silica gel). The product was dried in an oil-pump vacuum yielding allyl (4-methyl)coumarin-7-carbamate (0.75 g, 0.29 mmol, 34%) as a slightly yellow solid. *R*_f_ (SiO_2_, hexane : EtOAc 1 : 3, detection: UV): 0.72. ^1^H NMR (400 MHz, DMSO-d_6_): *δ*_H_ [ppm] 10.25 (1H, s), 7.70 (1H, d, *J* = 8 Hz), 7.55 (1H, d, *J* = 3 Hz), 7.41 (1H, dd, *J* = 8, 2 Hz), 6.24 (1H, s,), 6.05–5.95 (1H, m), 5.39 (1H, dd, *J* = 20, 4 Hz), 5.27 (1H, dd, *J* = 12, 4 Hz), 4.66 (1H, d, *J* = 4 Hz), 2.39 (3H, s). ^13^C NMR (100 MHz, DMSO-d_6_): *δ*_C_ [ppm] 161.1, 154.7, 152.9, 152.3, 141.6, 132.2, 125.5, 118.9, 115.8, 114.6, 113.4, 106.2, 66.5, 18.7. IR (ATR): *ν̃* [cm^−1^] 3271 (w), 1722 (m), 1685 (s), 1618 (s), 1583 (s), 1535 (m), 1423 (m), 1398 (m), 1353 (w), 1286 (w), 1232 (s), 1213 (m), 1172 (m), 1053 (s), 1020 (m), 937 (m), 844 (s), 819 (m), 750 (w), 721 (w), 709 (m). ESI MS (positive): *m*/*z* [%] 260.10 ([M + H]^+^, 20%), 282.20 ([M + Na]^+^, 20%).

### ^1^H NMR stability experiment

The ^1^H NMR stability experiment of the catalyst dissolved in deuterated DMSO was performed using a Bruker DRX-400 spectrometer at 298.5 K. Spectra were measured every 60 min for a period of 24 hours. The catalyst (4.5 mg, 8 μmol) was dissolved in 500 μL of d_6_-DMSO.

### Photochemical characterisation

DMSO (Uvasol, for spectroscopy, Merck) was commercially acquired and used without further purification. UV-Vis absorption spectra were recorded with a Cary 60 from Agilent Technologies, while the excitation and emission spectra were detected with a Fluorolog-3 from Horiba. The time-resolved emission experiments were performed with a home-built time-correlated single photon counting (TCSPC) setup using a pulsed 280 nm or 375 nm LED (Horiba) as excitation source, each yielding a time-resolution of about 1 ns (IRF) with the employed setup. The emission quantum yield was determined with an integrating sphere setup (Hamamatsu C9920-02). The concentration was set such that an optical density of 0.1 OD at the excitation wavelength was obtained for each sample.

### Medium for biological assays

Belitzky minimal medium (BMM)^71^ was used for cultivation of *B. subtilis* 168. The composition and sterilisation methods for the medium are summarised in Tables 3 and 4. The basal medium was autoclaved and stored separately from the supplements.

**Table 3:** Basal Medium (Belitzky minimal medium, BMM). Composition and sterilisation method. pH adjusted to 7.5 prior to autoclaving

Compound	Concentration in medium [mM]	Sterilisation method
(NH_4_)_2_SO_4_	15	Autoclaving
MgSO_4_·7H_2_O	8	
KCl	27	
Na_3_ citrate·2H_2_O	7	
Tris	50	

**Table 4:** Supplements. Composition and sterilisation method

Compound	Concentration in medium [mM]	Sterilisation method
KH_2_PO_4_	0.6	Sterile filtration
CaCl_2_·2H_2_O	2	
FeSO_4_·7H_2_O	0.001	
MnSO_4_·4H_2_O	0.01	
Glutamic acid	4.5	
l-Tryptophan	0.78	
Glucose	11	

### *In vitro* fluorescence catalysis experiments

Biologically-relevant conditions for the uncaging of 7-aminocoumarin-4-methane sulfonic acid, 2 and 7-amino-4-methylcoumarin, 4 were simulated. The culture medium (BMM) was used as solvent and the reactions were performed in the presence of an excess of air and thiols in form of glutathione. The absorption and emission maxima of the caged and free fluorophores were determined and can be extracted from Fig. 2.

To allow the calculation of the approximate yields of the catalytic uncaging reactions, the fluorescence intensities of different ratios of caged and free fluorophores were measured. To this end 200 μL BMM, 10 μL glutathione solution (10 mg mL^−1^), and 10 μL of different ratios of the free and protected dyes (1 mg mL^−1^) were mixed and added to a microtiter plate (*Greiner Bio-One*, 96-well plate (chimney well), black with clear bottom, non-binding).

For catalysis experiments, 200 μL BMM, 10 μL glutathione solution (10 mg mL^−1^), and 10 μL of the caged fluorophore (1 mg mL^−1^) were mixed and added into microplate wells. At this point, the catalyst was dissolved in DMSO and added. The samples were excited at 357 nm in the case of 7-aminocoumarin-4-methanesulfonic acid and 356 nm in the case of 7-amino-4-methylcoumarin. The increase in fluorescence intensity was recorded at 37 °C every 2 min for 6 hours and the plate shaken before each measurement.

### Determination of the ruthenium content in bacteria by ICP-OES

Samples for ICP-OES analysis were prepared as described before.^72^ For the entire experiment of the determination of the ruthenium content of bacterial cells, only metal-free plastic ware and ultrapure water (Bernd Kraft, Duisburg, Germany) were used. *B. subtilis* 168 cultures were grown in 50 mL of BMM to early exponential growth phase and the cultures were treated with 1 μg mL^−1^ catalyst or left untreated (negative control). OD_500_ values at the start of the incubation are given in Table 5. After 15 min of treatment with the catalyst, cells were harvested by centrifugation (4000× *g*, 4 °C, 5 min), washed twice in 100 mM tris/1 mM EDTA, pH 7.5, and washed once in 100 mM tris, pH 7.5. Cells were disrupted in 500 μL of 100 mM tris, pH 7.5 by ultrasonication on ice (8 cycles of 1 min pulse with 1 min pause, 90% amplitude, cycle 0.5) using a VialTweeter (Hielscher, Teltow, Germany). Debris and soluble fractions were separated *via* centrifugation (20 000 × *g*, 4 °C, 20 min) and the soluble fractions were dried. Subsequently, the pellets were completely dissolved in 2.5 mL 65% nitric acid (Bernd Kraft, Duisburg, Germany) and incubated at 80 °C for 16 hours. Dissolved samples were made up to 10 mL with ultrapure water (Bernd Kraft, Duisburg, Germany). Ruthenium concentrations were determined by inductively coupled plasma optical emission spectroscopy using an iCAP* 6300 Duo View ICP Spectrometer (Thermo Fisher Scientific, Waltham, MA, USA). Liquid calibration standards from 10 μg L^−1^ to 10 mg L^−1^ of ruthenium (Bernd Kraft, Duisburg, Germany) were also analysed. The resulting element concentrations were converted into intracellular ruthenium concentrations based on the number of cells harvested (an OD_500_ of 1 corresponds to 6 × 107 cells) and the cytosolic volume of *B. subtilis*. This volume was taken as 3.09 × 10^−9^ μL based on average rod size of *B. subtilis* determined by cry-electron microscopy by Matias and Beveridge.^73,74^

**Table 5:** Optical density (OD_500_) of samples used for determination of Ru content

Sample	OD_500_
Control 1	0.48
Control 2	0.36
Control 3	0.41
Catalyst 1	0.52
Catalyst 2	0.43
Catalyst 3	0.44

### Effect of the catalyst and the fluorophores on cells

*B. subtilis* 168 cultures were grown at 37 °C under steady agitation in BMM. The cultures were grown to an optical density at 500 nm (OD_500_) of 0.35, split, and exposed to the catalyst Ru3 (at the following concentrations: 0.05 μg mL^−1^, 0.1 μg mL^−1^, 0.2 μg mL^−1^, 0.4 μg mL^−1^, 0.6 μg mL^−1^, 1 μg mL^−1^, and 5 μg mL^−1^), or to the fluorophores 1–4 (at the following concentrations: 10 μg mL^−1^, 25 μg mL^−1^, and 50 μg mL^−1^). Untreated cultures served as a negative control. The OD_500_ was measured every 30 min for 2 h and then after 3 h and 4 h.

### Incubation of bacteria with caged dye and subsequent catalyst addition

*B. subtilis* 168 cultures were grown in BMM to an OD_500_ of 0.35 and subsequently treated with 10 μg mL^−1^, 25 μg mL^−1^, and 50 μg mL^−1^ of 2 and 4. After 15 min of exposure to the caged fluorophores, the cultures were harvested by centrifugation (4000 × *g*, 4 °C, 5 min), washed with 200 μL of BMM, and resuspended in 1 mL BMM. For the emission measurements with a microplate reader, 200 μL of the prepared bacterial cultures were transferred to a 96-well microplate (Greiner Bio One, 96-well plate (chimney well), black with clear bottom, non-binding). The microplates were placed in a plate reader (Tecan, Infinite M Nano+) to monitor the fluorescence intensities of the cultures in the wells. At this point, Ru3 was dissolved in DMSO and added to the wells at a concentration of 1 μg mL^−1^. Two negative controls were performed during the assay: cultures not exposed to 2, 4, or Ru3, and cultures exposed to the fluorophores, but not Ru3. The absorption and emission wavelengths for the different fluorophores can be extracted from Fig. 3. The plates were maintained at 37 °C and shaken for 5 s before the measurement. The absorbance and the fluorescence intensity of each well were read every 2 min for 4 hours.

### Retention of uncaged dyes inside *B. subtilis* cells

*B. subtilis* cultures were grown in BMM to an OD_500_ of 0.35 and subsequently treated with 50 μg mL^−1^ of 2 or 4. After 15 min of exposure to the caged fluorophores, the cultures were harvested by centrifugation (4000*g*, 4 °C, 5 min) and washed twice with 800 μL of BMM. The solution from the first wash was discarded as it visibly contained leftover dye from outside the bacteria and the vials. A second wash was incubated for 15 min to allow for equilibration. After the second wash, the supernatant was filtered using glass microfiber filters (Whatman Mini-Uniprep™, pore size 0.45 μm) and 200 μL of the filtrate were transferred to a 96-well microplate (Greiner Bio One, 96-well plate (chimney well), black with clear bottom, non-binding). The cell pellet was dissolved in 400 μL of 100 mM tris, pH 7.5, and the cells were disrupted by ultrasonication on ice (8 cycles of 1 min pulse with 1 min pause, 90% amplitude, cycle 0.5) using a VialTweeter (Hielscher, Teltow, Germany). Debris and soluble fractions were separated by centrifugation (20 000 × *g*, room temperature, 10 min) and 200 μL of the soluble fractions (crude extracts) were transferred to a 96-well microplate for fluorescence measurements as described above. At this point, Ru3 was dissolved in DMSO and added to the wells at a concentration of 1 μg mL^−1^. Two negative controls were performed in this assay: Samples from untreated cultures and cultures treated only with the caged fluorophores were analysed in parallel experiments. The plates were maintained at 37 °C and shaken for 5 s before each measurement. The absorbance and the fluorescence intensity of each well were read every 2 min for 4 hours.

### Localisation of caged dye compared to its uncaged version inside *B. subtilis* cells

*B. subtilis* 168 cultures were grown in BMM to an OD_500_ of 0.35 and subsequently treated with 50 μg mL^−1^ of 4. After 15 min of exposure to the caged fluorophore, the cultures were harvested by centrifugation (4000 × *g*, 4 °C, 5 min) and washed twice with 800 μL of BMM. The cultures were resuspended in 800 μL of BMM. Subsequently, the catalyst Ru3 was dissolved in DMSO and added to the cultures at a concentration of 1 μg mL^−1^. The cultures were incubated for 2 hours at 37 °C under steady agitation followed by harvesting the cells by centrifugation (13 200*g*, room temperature, 10 min). The supernatant was filtered using glass microfiber filters (Whatman Mini-Uniprep, pore size 0.45 μm) and 200 μL of the filtrate were transferred to a 96-well microplate. The cell pellet was dissolved in 400 μL of 100 mM tris, pH 7.5, and the cells were disrupted by ultrasonication on ice (8 cycles of 1 min pulse with 1 min pause, 90% amplitude, cycle 0.5). Debris and soluble fractions were separated by centrifugation (20 000 × *g*, room temperature, 10 min) and 200 μL of the soluble fractions were transferred to 96-well microplates for fluorescence measurements. Data is shown as relative % amounts of the caged dye (4) in each fraction and uncaged dye (3) in each fraction, following Ru3 addition, Fig. 8. The relative amount of 4 and 3 cannot be determined, as extinction coefficients weren’t established. Raw fluorescence intensities for these measurements including no catalyst controls, can be found in Fig. S14 (ESI†).

### Incubation of bacteria with caged dye and subsequent addition of various siderophore-conjugated catalysts

For these reactions, the composition of BMM was modified. Instead of sodium citrate (7 mM), sodium chloride (20 mM) was used in the basal medium. Otherwise, composition and sterilisation methods remain as given above in Tables 3 and 4. The citrate-free BMM was autoclaved and stored separately from the supplements. The following experiments were performed in this citrate-free BMM in parallel with and without the addition of the supplemental iron to the basal medium. The experiments were performed in technical triplicates.

*B. subtilis* cultures were grown in citrate-free BMM (with and without Fe) to an OD_500_ of 0.5 and subsequently treated with the protected fluorophore 4 at a concentration of 50 μg mL^−1^. After 15 min of exposure to the fluorophores, the cultures were harvested by centrifugation (4000 × *g*, 4 °C, 5 min), washed with 200 μL BMM, and resuspended in 1 mL of the same medium. For the emission measurements with a microplate reader, 200 μL of the prepared bacterial cultures were transferred to a 96-well microplate (Greiner Bio-One, 96 well plate [chimney well], black with clear bottom, non-binding). The microplates were placed in a plate reader (Tecan, *Infinite M Nano+*) to monitor the fluorescence intensities of the cultures in the wells. At this point, the different catalysts were dissolved in DMSO and added to the wells at a concentration of 1 μg mL^−1^. Two negative controls were performed during the assay: untreated cultures and cultures only treated with 4, but no Ru catalyst. The excitation and emission wavelength for the fluorophore is shown in Table 6. The plates were maintained at 37 °C and shaken for 5 s before each measurement and the fluorescence intensity of each well was recorded every 2 min for 4 h at the emission maximum of 7-amino-4-methylcoumarin 3.

**Table 6:** Excitation and emission wavelengths for 3 and 4 used in the uncaging experiments for siderophore-linked Ru catalysts Ru4–Ru7

Fluorophore	Excitation maximum [nm]	Emission maximum [nm]
7-Amino-4-methylcoumarin (3)	356	421
Alloc-7-amino-4-methylcoumarin (4)	329	392

### Statistical analysis

Cell culture experiments were performed once. The bioorthogonal uncaging (photometric) assays were performed in three biological replicates starting from three independent overnight cultures, with which experiments were performed and measurements carried out on three different days (samples for ICP-MS analysis were collected and processed on the same day). Mean values and standard deviations were calculated from these triplicate repetitions and used to generate graphs/tables.

## Abbreviations

Alloc: Allylcarbamate
*B. subtilis*
: *Bacillus subtilis* 168
BMM: Belitzky minimal medium
ICP-OES: Inductively coupled plasma optical emission spectrometry
OD: Optical density
o.n.: Over night
RFU: Relative fluorescence units
TMC: Transition metal catalyst
TON: Turnover number
TCSPC: Time-correlated single-photon counting

## Author contributions

Nicole Schubert: methodology, investigation, visualisation, writing – original draft; James W. Southwell: investigation (siderophore-conjugated Ru catalysts), writing – review and editing; Melissa Vázquez-Hernández: investigation, resources (microbiology), writing – review and editing; Svenja Wortmann: photophysical investigations, visualisation, writing; Sylvia Schloeglmann: photophysical investigations, writing – review and editing; Anne-Kathrin Duhme-Klair: supervision, writing – review and editing, funding acquisition; Patrick Nuernberger: supervision, writing – review and editing; Julia E. Bandow: supervision, writing – review and editing; Nils Metzler-Nolte: conceptualisation, supervision, writing – review and editing, funding acquisition.

## Conflicts of interest

The authors declare no conflict of interest.

## Supplementary Material

CB-005-D4CB00187G-s001

## Data Availability

The authors confirm that all data are available as ESI.† Furthermore, additional data and original files are available from the authors upon reasonable request.
