# Supplementary material for: Fluorescent probes for investigating the internalisation and action of bioorthogonal ruthenium catalysts within Gram-positive bacteria
Source: RSC Chem Biol. 2024 Oct 15;5(12):1201–13. doi: 10.1039/d4cb00187g (PMC11477652; doi:10.1039/d4cb00187g)
Supplement: CB-005-D4CB00187G-s001 [file CB-005-D4CB00187G-s001.pdf]

Supplementary Information (SI) for RSC Chemical Biology.
This journal is © The Royal Society of Chemistry 2024

Electronic Supporting Information

Fluorescent Probes for Investigating the Internalisation and Action of Bioorthogonal Ruthenium Catalysts within Gram-positive Bacteria

Nicole Schubert[a, ‡], James W. Southwell[b, ‡], Melissa Vázquez-Hernández[c], Svenja Wortmann[d], Sylvia Schloeglmann[d], Anne-Kathrin Duhme-Klair[b], Patrick Nuernberger[d], Julia E. Bandow[c], Nils Metzler-Nolte[*a]

a *Faculty of Chemistry and Biochemistry, Chair of Inorganic Chemistry I – Bioinorganic Chemistry, Ruhr University Bochum, Universitätsstraße 150, 44801 Bochum, Germany*

b *Department of Chemistry, University of York, Heslington, York, YO10 5DD, UK*

c *Faculty of Biology and Biotechnology, Applied Microbiology, Ruhr University Bochum, Universitätsstraße 150, 44801 Bochum, Germany*

d *Institut für Physikalische und Theoretische Chemie, Universität Regensburg, Universitätsstraße 31, 93053 Regensburg, Germany*

‡ These authors contributed equally

* Corresponding author email address: nils.metzler-nolte@rub.de

Contents

1. **Syntheses**
    a. *Synthesis of* ***Ru3***
    b. *Synthesis of 8-hydroxyquinoline-5-carboxylic acid*
2. **Photophysical Characterisation of Fluorophores**
3. **^{1}H NMR and ^{13}C NMR Spectra**
    a. ***Ru3*** *in DMSO over time*
    b. *Comparison of allyl alcohol and* ***Ru3*** *dissolved in DMSO*
    c. *Caged 7-aminocoumarin-4-methanesulfonic acid,* ***2***
    d. *Caged 7-Amino-4-methylcoumarin,* ***4***
4. **Catalytic *in vitro* Activity of Ru3**
5. **Effects of Ru3 and Fluorophores 1-4 on *B. subtilis* growth**
6. **Localisations of the Uncaged and Caged Dye in *B. subtilis***

1. Syntheses

a. Synthesis of ***Ru3***

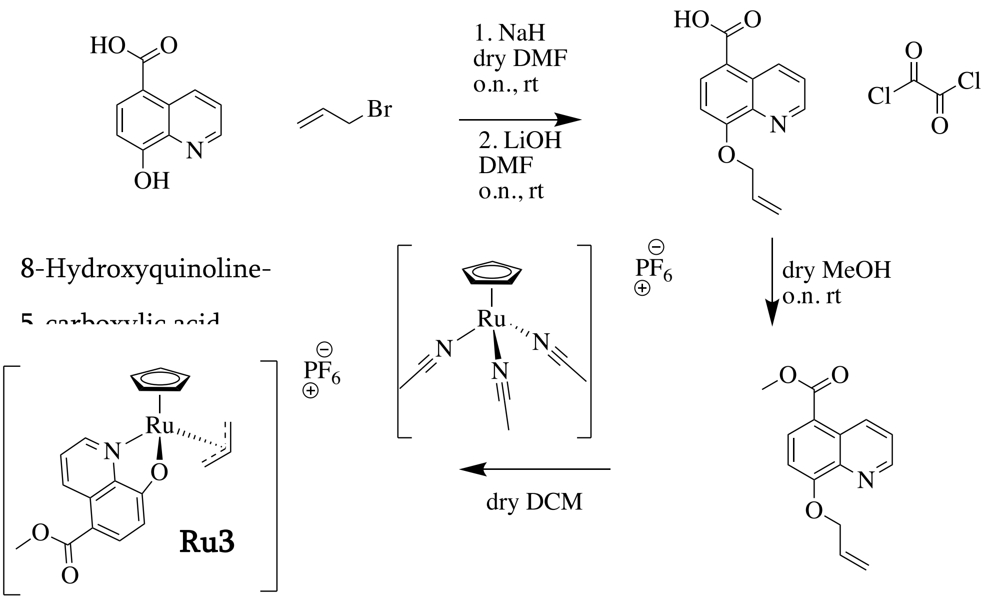

Figure S 1 Synthesis of **Ru3** based on the procedure published by Meggers *et al.*[1]

b. Synthesis of 8-Hydroxyquinoline-5-carboxylic acid

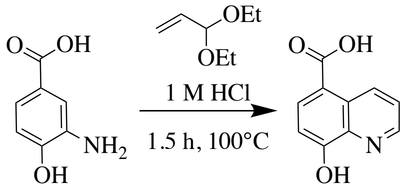

Figure S 2 Synthesis of 8-hydroxyquinoline-5-carboxylic acid from 4-hydroxy-3-aminobenzoic acid.[31]

2. Photophysical Characterisation of Fluorophores

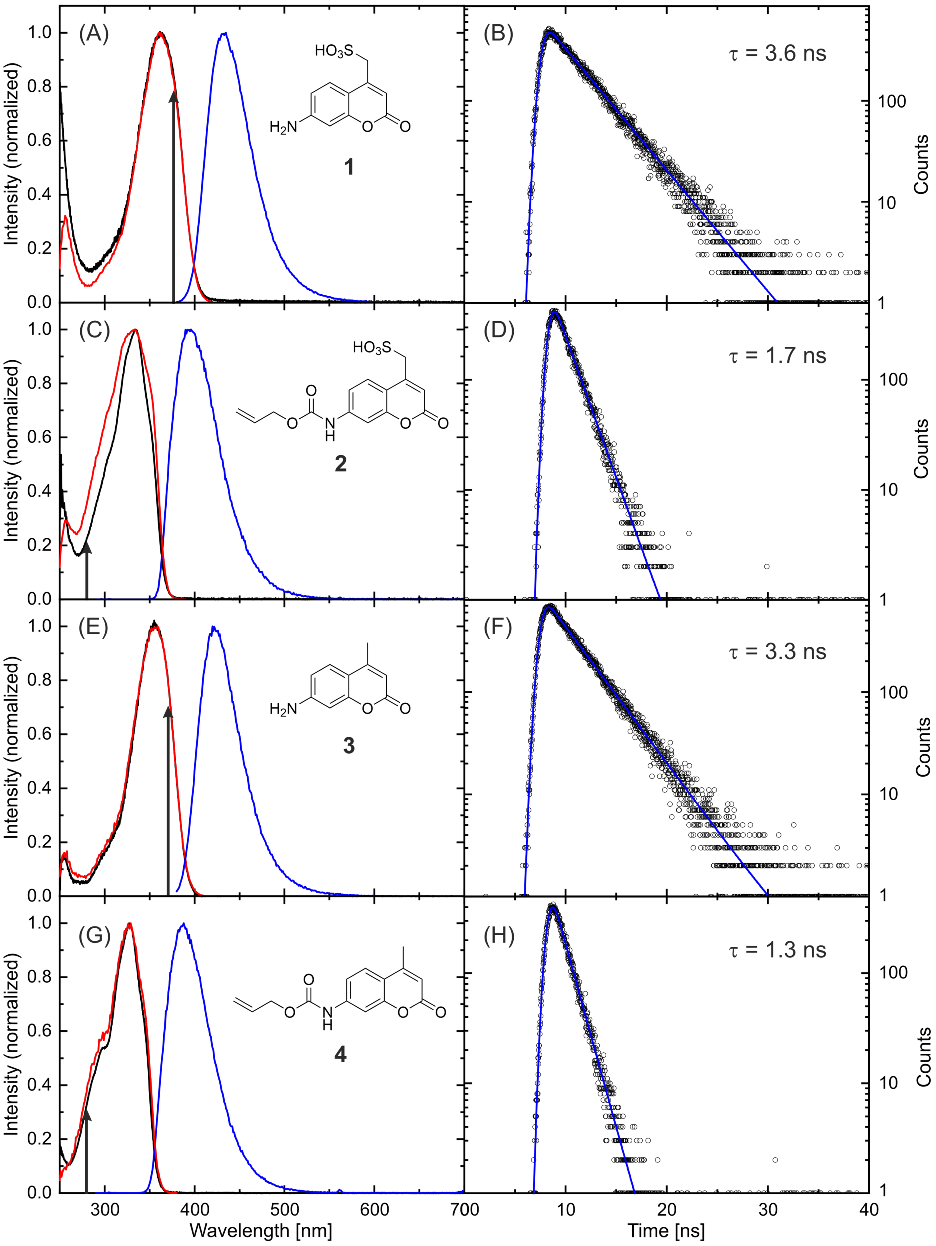

Figure S 3 Normalised absorption (black), excitation (red) and emission spectra (blue) of the two coumarin pairs, **1/2** and **3/4** dissolved in DMSO. The excitation wavelength to detect the corresponding steady-state emission spectrum and the time-resolved emission decay is indicated by the black arrows in each UV-Vis spectrum, i.e., 370 nm for the unsubstituted dyes, **1** and **3**, as well as 280 nm in the case of the substituted fluorophores **2** and **4**. The excitation spectra correspond to the respective emission maximum, i.e., 430 nm for **1**, 400 nm for **2**, 420 nm for **3** and 390 nm for **4**. The right panel shows the time-resolved emission traces detected at the emission maximum (black dots) with a TCSPC setup and the corresponding monoexponential fit (blue line).

3. ^{1}H NMR and ^{13}C NMR Spectra

*a. **Ru3** in DMSO over time*

Since **Ru3** is highly soluble in this solvent, we started our investigation by analysing the catalyst's stability in DMSO, as there are concerns for the stability of this system.[35] To this end, **Ru3** was dissolved in deuterated DMSO and ^{1}H NMR spectra were recorded in intervals of 60 min for 24 hours. Selected spectra are shown in **Figure S 4**.

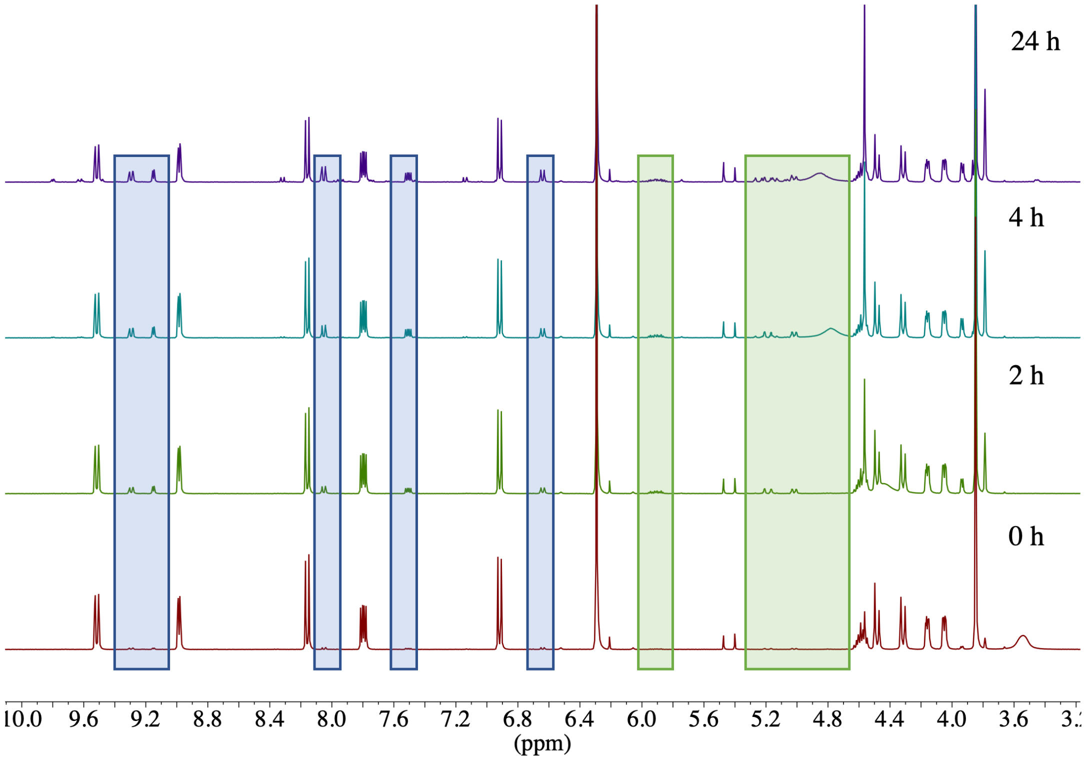

Figure S 4 Changes in the ^{1}H NMR spectra of **Ru3** over time in deuterated DMSO. The green and blue boxes highlight two new sets of signals that arise after dissolving **Ru3** in deuterated DMSO.

After two hours, two new sets of resonances emerged, highlighted in green and blue boxes. The set of signals shown in green boxes corresponds to the free allyl alcohol (see also Figure S4, which confirms exact matching of these signals with free added allyl alcohol). This observation is in line with the catalytic cycle proposed by Meggers *et al.*,[2] as generation of the active Ru^{II} species results from attack by nucleophiles such as thiols, or in this case residual water in the DMSO. It is hypothesised that the vacant coordination site produced is then occupied by DMSO. The formation of a DMSO complex is consistent with the appearance of a new set of signals in the aromatic region, marked with blue boxes.

*b. Comparison of allyl alcohol and **Ru3** dissolved in DMSO*

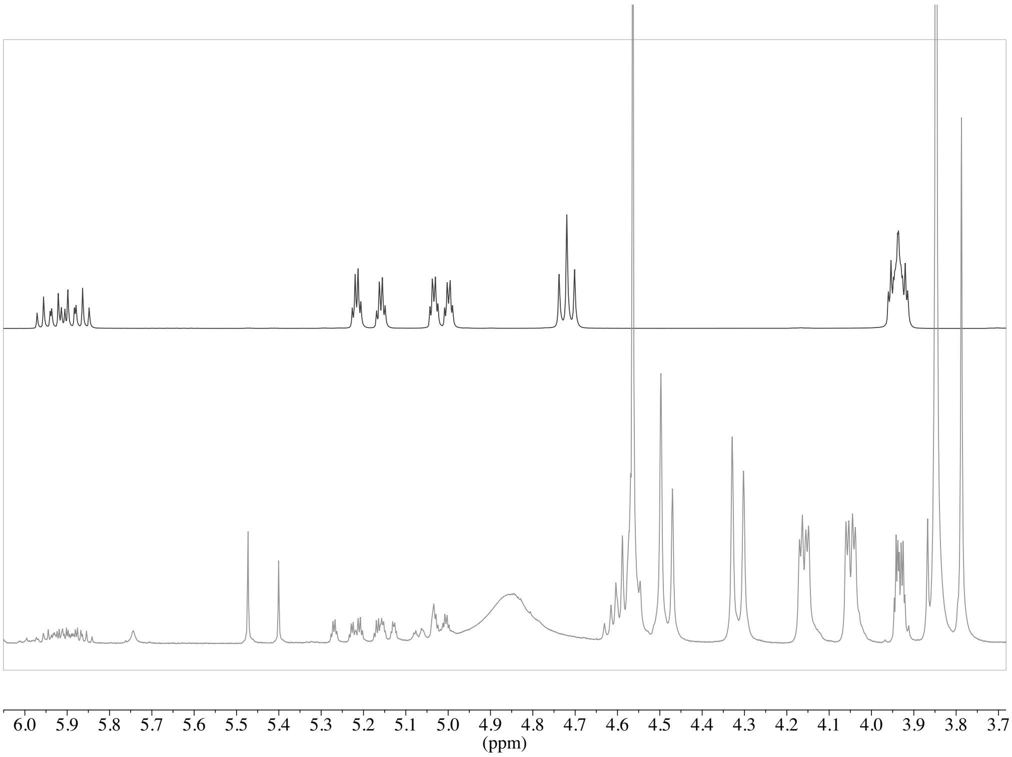

Figure S 5 Comparison of the ^{1}H spectrum of free allyl alcohol (top) measured in *deuterated* DMSO and the catalyst **Ru3** (bottom) dissolved in *deuterated* DMSO after 10 h.

*c. Caged 7-aminocoumarin-4-methanesulfonic acid, **2***

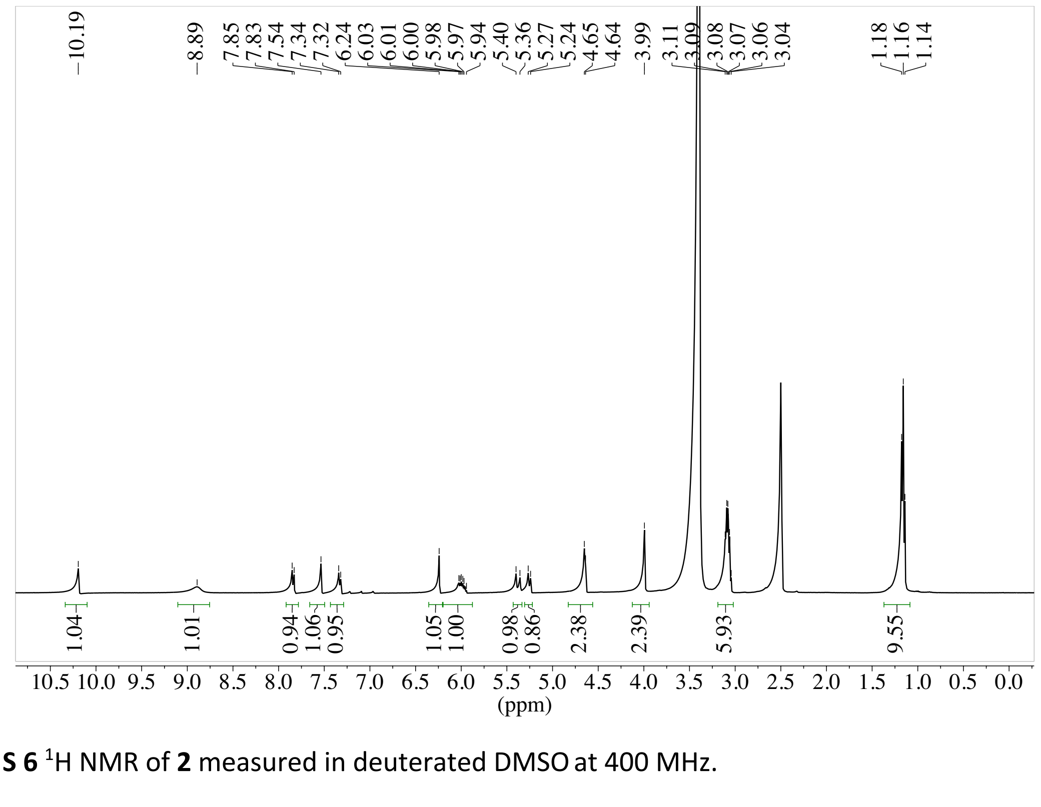

Figure S 6 ^{1}H NMR of **2** measured in deuterated DMSO at 400 MHz.

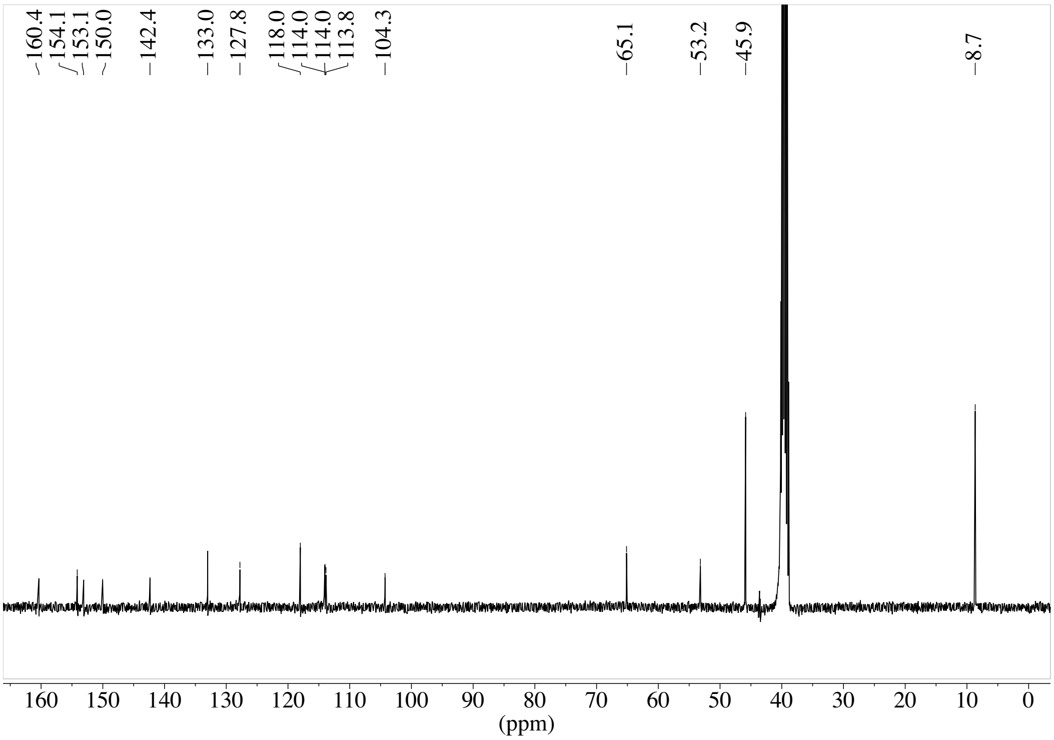

Figure S 7 ^{13}C NMR of **2** measured in deuterated DMSO at 100 MHz.

*d. Caged 7-amino-4-methylcoumarin, **4***

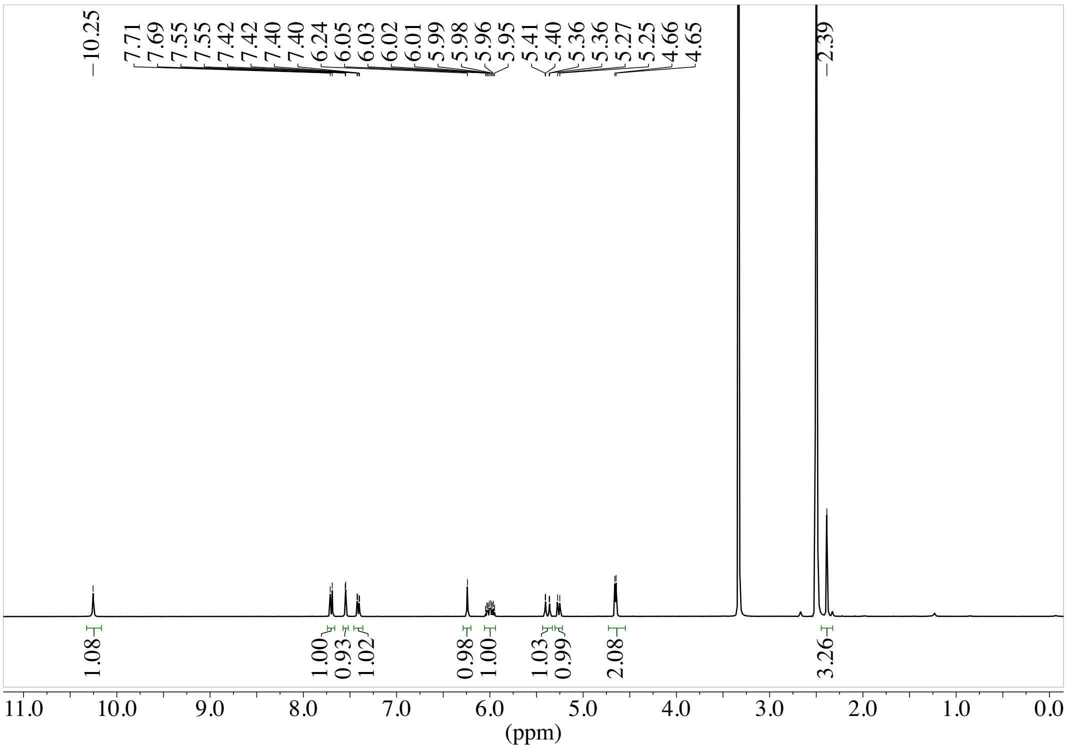

Figure S 8 ^{1}H NMR of **4** measured in deuterated DMSO at 400 MHz.

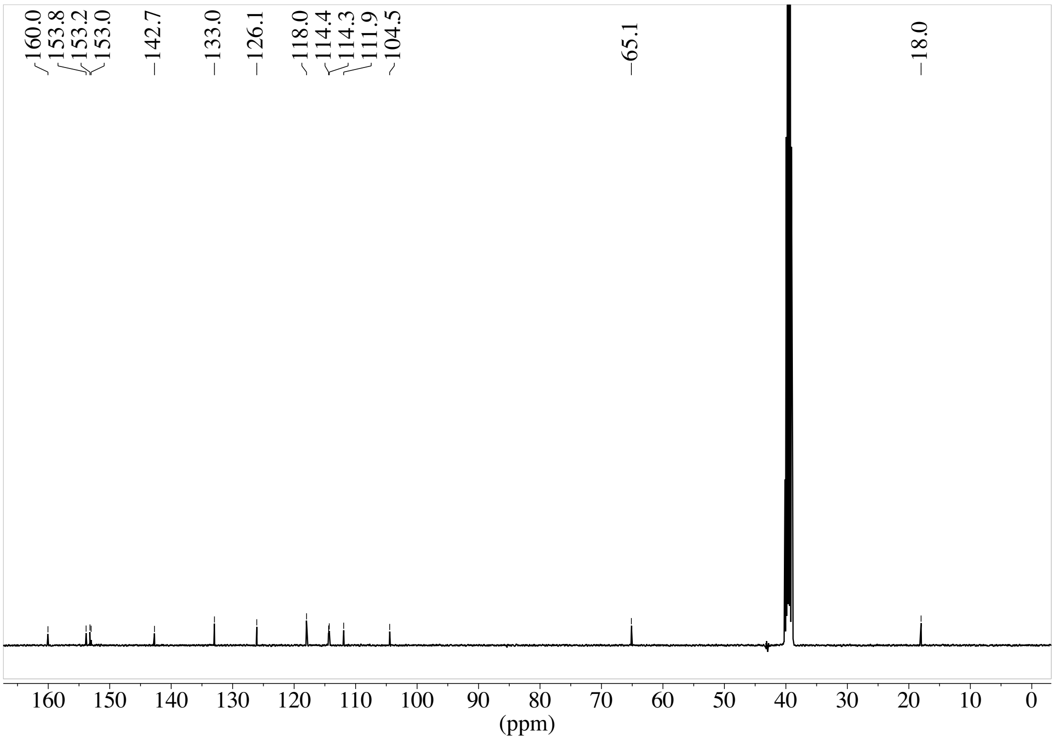

Figure S 9 ^{13}C NMR of **4** measured in deuterated DMSO at 100 MHz.

4. Catalytic *in vitro* Activity of Ru3

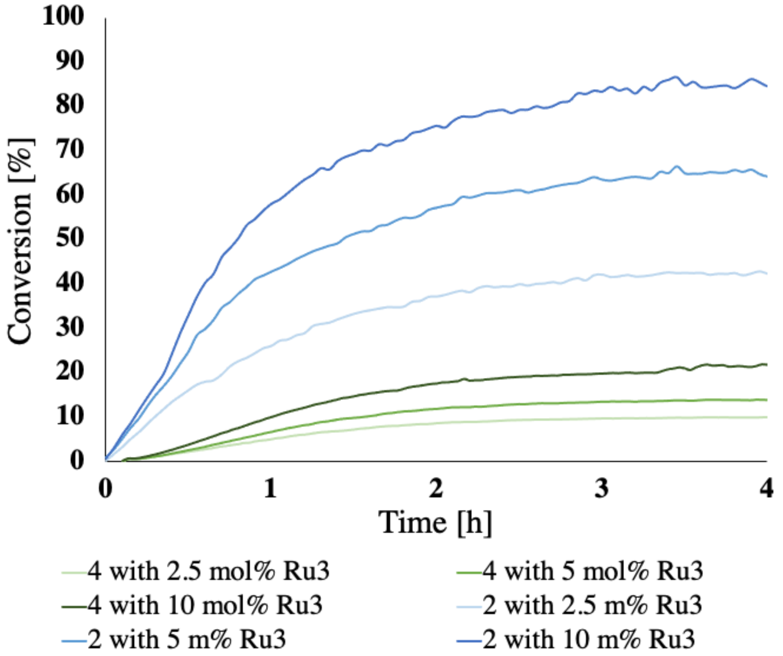

Figure S 10 Conversion for the *in vitro* deprotection of **2** and **4**, respectively, using **Ru3** in different mol%.

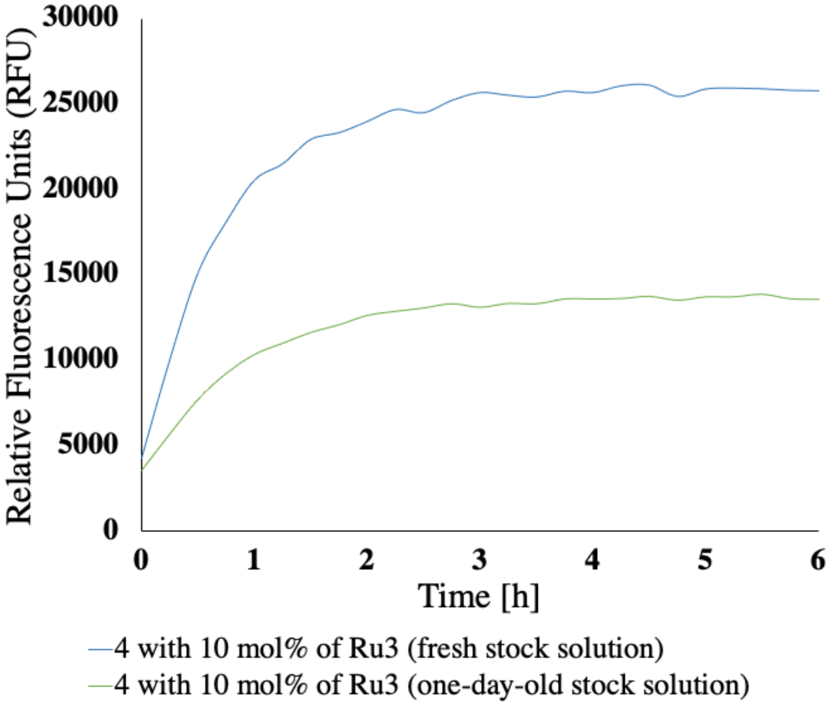

Figure S 11 Comparison of the increase in fluorescence intensity for the masked 7-amino-4-methyl coumarin, **4** when a fresh (blue curve) and a one-day-old solution (green curve) of the catalyst dissolved in DMSO were used.

5. Effects of Ru3 and Fluorophores 1-4 on *B. subtilis* Growth

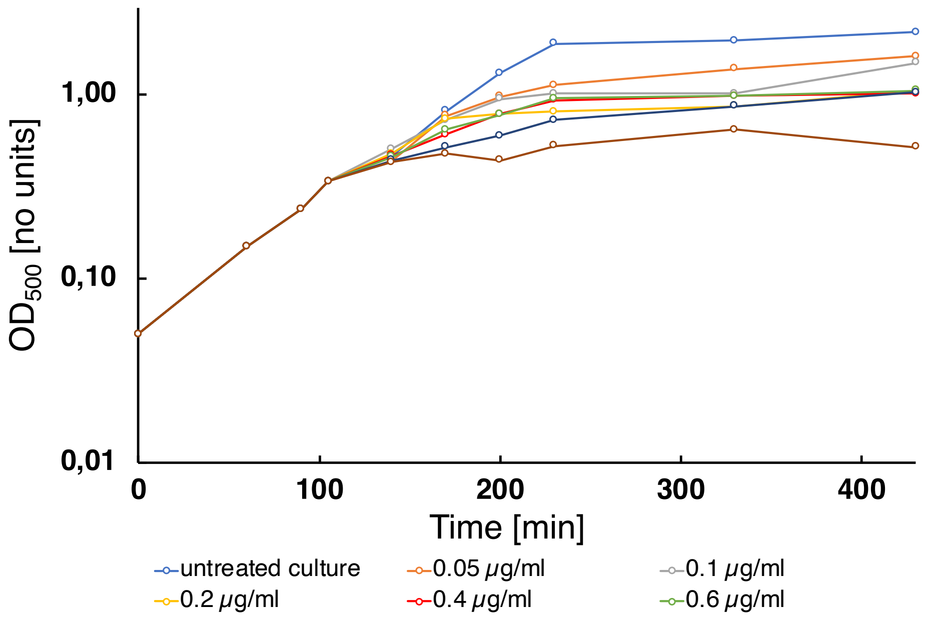

Figure S 12 Cultures of *B. subtilis* were incubated with different concentrations of **Ru3**. The untreated culture shown in blue served as growth control.

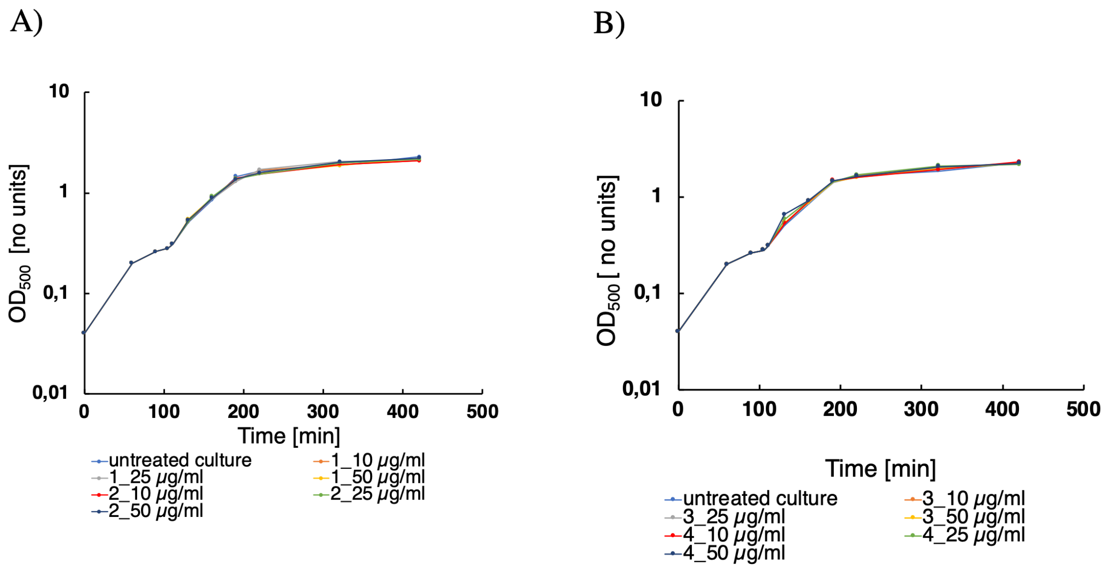

Figure S 13 Cultures of *B. subtilis* were incubated with different concentrations of the fluorophores **1**-**4**. An untreated culture served as growth control. Graph A) shows the growth behaviour of cultures treated with 7-aminocoumarin-4-methanesulfonic acid, **1** and its masked derivative, **2** and graph B) shows the growth behaviour of cultures treated with 7-amino-4-methylcoumarin, **3** and its masked derivative, **4**.

6. Localisations of the Uncaged and Caged Dye in *B. subtilis*

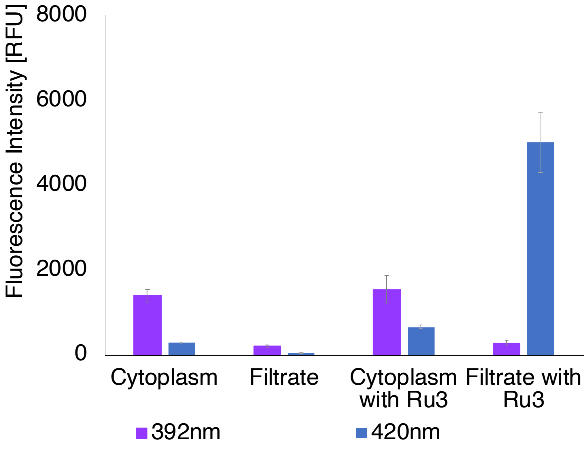

Figure S 14 Measured fluorescence intensity of cytoplasmic and extracellular fractions following incubation of *B. subtilis* with **4** and addition of **Ru3** (or DMSO, for no catalyst controls), 2 hours before harvest. The fluorescence intensities at 392 nm (emission maximum of **4, violet**) and 420 nm (emission maximum of **3, blue**) were measured using a quarter of each filtrate sample and half of each lysate sample volume. The error bars are the standard deviations of three independent biological replicates.
